# mTORC2 regulates auditory hair cell structure and function

**DOI:** 10.1016/j.isci.2023.107687

**Published:** 2023-08-19

**Authors:** Maurizio Cortada, Soledad Levano, Michael N. Hall, Daniel Bodmer

**Affiliations:** 1 Department of Biomedicine, University of Basel, CH-4031 Basel, Switzerland; 2 Biozentrum, University of Basel, CH-4056 Basel, Switzerland; 3 Clinic for Otorhinolaryngology, Head and Neck Surgery, University of Basel Hospital, CH-4031 Basel, Switzerland

**Keywords:** Molecular biology, Cell biology

## Abstract

mTOR broadly controls cell growth, but little is known about the role of mTOR complex 2 (mTORC2) in the inner ear. To investigate the role of mTORC2 in sensory hair cells (HCs), we generated HC-specific *Rictor* knockout (HC-RicKO) mice. HC-RicKO mice exhibited early-onset, progressive, and profound hearing loss. Increased DPOAE thresholds indicated outer HC dysfunction. HCs are lost, but this occurs after hearing loss. Ultrastructural analysis revealed stunted and absent stereocilia in outer HCs. In inner HCs, the number of synapses was significantly decreased and the remaining synapses displayed a disrupted actin cytoskeleton and disorganized Ca^2+^ channels. Thus, the mTORC2 signaling pathway plays an important role in regulating auditory HC structure and function via regulation of the actin cytoskeleton. These results provide molecular insights on a central regulator of cochlear HCs and thus hearing.

## Highlights

• mTORC2 knockout (KO) in sensory hair cells (HCs) leads to profound hearing loss
• HC loss occurs only secondary to hearing loss in mTORC2 KO mice
• mTORC2 KO in HCs leads to stereocilia shortening and synaptic defects
• mTORC2 KO in HCs reduces synaptic actin cytoskeleton and disorganizes Ca^2+^ channels

## Graphical abstract

**Figure:**
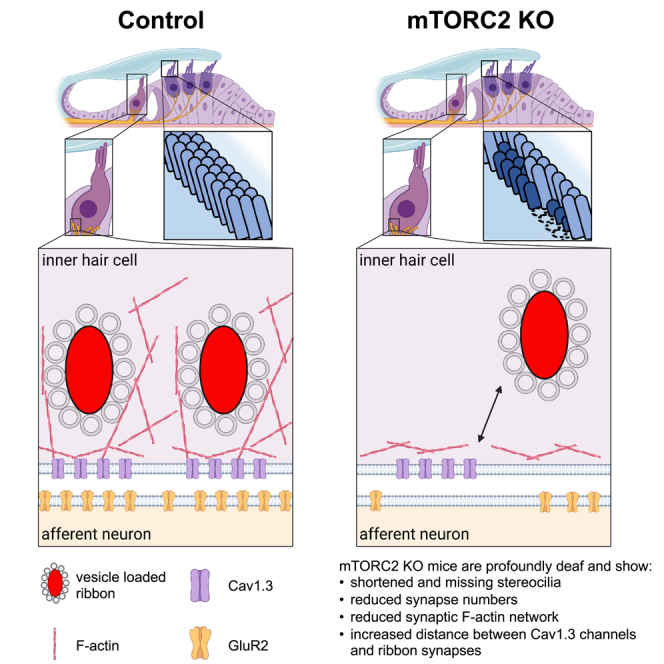

## Introduction

Sensorineural hearing loss affects a significant and increasing proportion of the population. It is the third most important cause of years lived with disability^1^ and the World Health Organization (WHO) estimates that around 5.5% of the global population suffers from disabling hearing loss that requires rehabilitation.^2^ Due to an increase in life expectancy and other factors such as exposure to environmental noise, this number is expected to increase to over 7% by 2050.^2^ However, despite the significant societal burden of hearing loss, there are no adequate treatment options for hearing loss other than prosthetic devices. Most hearing loss, both genetic and non-genetic, has an underlying defect in the peripheral hearing organ, the cochlea.^3^^,^^4^ To date, 124 non-syndromic hearing loss genes (with hearing loss as the only symptom)^5^ and around 400 genes for syndromic hearing loss^3^ have been identified. It is expected that an additional 350–400 genes are required for hearing.^3^ The discovery of hearing loss genes contributed significantly to our understanding of the molecular composition and function of the hearing organ, especially the sensory cells, which are few in number and thus biochemically inaccessible. Less is known about signaling pathways that regulate sensory cell function in the inner ear.

Sound is perceived by sensory cells, called hair cells (HCs), in the cochlea of the inner ear. HCs in the vestibule of the inner ear mediate balance. In the mammalian cochlea, there are two types of HCs. Inner hair cells (IHCs), present in one row along the cochlear spiral, are the actual sensory cells. They transmit all the auditory information to the brain via synaptic contacts on afferent fibers of the auditory nerve. Outer hair cells (OHCs), present in three rows, are electromotile to increase sensitivity and frequency selectivity. Depending on their place along the cochlear spiral, HCs respond to different frequencies. High frequencies are perceived at the base of the cochlea whereas lower frequencies at the apex. HCs have microvilli-like protrusions, a so-called hair bundle, on their apical side. These protrusions, also known as stereocilia, are the sound and motion sensors. Deflection of the stereocilia, induced either by sound or head motion, opens mechanoelectrical transduction (MET) channels. This depolarizes HCs leading to electromotility or synaptic transmission onto the vestibulocochlear nerve which relays the information to the central nervous system.

HC loss often correlates with and is thus considered the major cause of hearing loss. However, noise exposure or aging can cause loss of synapses without loss of HCs, a phenomenon termed cochlear synaptopathy.^6^^,^^7^ Loss of HCs and synapses is permanent; adult cochlear HCs do not regenerate in mammals. In addition, there are no specific molecular or cellular therapies for hearing loss. Today, sensorineural hearing loss can be addressed only with hearing aids or cochlear implants. Detailed molecular understanding of HC function and dysfunction is needed to develop better preventive and therapeutic strategies.

Mammalian/mechanistic Target of Rapamycin (mTOR) is a serine/threonine kinase that regulates many cellular processes as part of two structurally and functionally distinct complexes, mTOR complex 1 (mTORC1) and mTORC2. Due to the availability of the specific inhibitor rapamycin, the function of mTORC1 has been investigated extensively. It is a master regulator of cell growth, promoting anabolism and inhibiting catabolic processes such as autophagy. Increasing evidence suggests a damaging role of mTORC1 overactivation in the cochlea, whereas mTORC1 inhibition in the neurosensory epithelium promotes HC survival and hearing protection^8^ (and reviewed in^9^). In contrast to mTORC1, mTORC2 is insensitive to acute rapamycin treatment and is less well studied. mTORC2 is found at different subcellular localizations to regulate various cellular processes including actin cytoskeletal organization.^10^^,^^11^^,^^12^

The rapamycin-insensitive companion of mTOR (RICTOR) is an essential and specific subunit of mTORC2 and its disruption has been widely used to study mTORC2 signaling. The major mTORC2 substrates are members of the AGC kinase family, including Akt, protein kinase C (PKC), and serum and glucocorticoid-induced kinase 1 (SGK1).^13^^,^^14^ Only a few studies relate PKC or SGK1 signaling to HC function. PKC alpha has been observed in HCs^15^ and linked to IHC synaptic protein regulation^16^ and OHC electromotility.^17^ Moreover, PKC isoforms have been linked to postsynaptic functions in HCs.^18^^,^^19^^,^^20^ For SGK1, no specific function has yet been described in mammalian HCs, but it has been implicated in ototoxicity.^21^ In contrast, phosphoinositide 3-kinase (PI3K)/Akt signaling is better studied in the cochlea and widely appreciated as an HC survival pathway (reviewed in^9^). The pro-survival role of Akt activation in HCs has been shown using phosphorylation of Ser473 in the Akt hydrophobic motif as a readout. mTORC2 phosphorylates Ser473 to achieve maximal Akt activation.^22^^,^^23^ In a recent study, *Rictor* deletion in both HCs and surrounding supporting cells of the inner ear at an early developmental stage (E14.5) resulted in hearing loss caused by rapid HC death.^24^ Mechanistically, it was proposed that this is mainly due to loss of Akt signaling.^24^ However, *Akt1* knockout (KO) and *Akt2, Akt3* double KO mice, which display hearing loss, do not show any sign of HC loss or other cellular defects in the cochlea.^25^ Therefore, the precise role of mTORC2 in sensory HCs remains incompletely understood. To better understand the role of mTORC2 specifically in sensory HCs of the inner ear, we generated HC-specific *Rictor* KO mice. Our study shows that loss of *Rictor* leads to profound hearing loss due to abnormal stereocilia, reduced synaptic cytoskeleton, disorganization of synaptic proteins and loss of synapses, rather than HC death. Thus, this study elucidates the role of mTORC2 signaling in regulating HC structure and function.

## Results

### Generation of hair cell specific *Rictor* knockout mice

**Figure 1:**
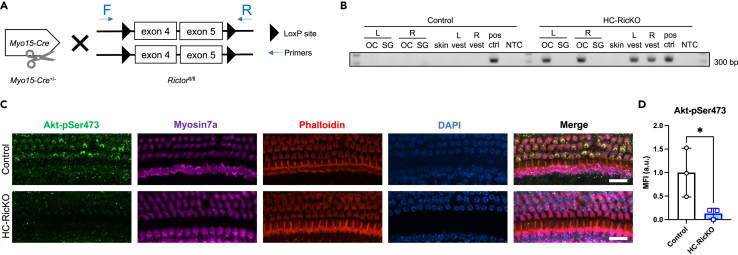
KO confirmation of HC-RicKO mice (A) Hemizygous *Myo15*-*Cre*^+/−^ mice were crossed with homozygous *Rictor*^fl/fl^ mice harboring *LoxP* sites upstream and downstream of exon 4 and 5, respectively. Forward (F) and reverse primers (R) were used to detect Cre-mediated recombination, resulting in a 280 bp PCR product after Cre-mediated recombination of the *Rictor* allele. (B) PCR using genomic DNA from dissected organ of Corti (OC), spiral ganglion (SG) or vestibular organ (vest; macular and cristae ampullaris organs pooled) tissue, separate for left (L) and right (R) inner ears. Genomic DNA from outer ear skin of both genotypes was used as negative control of Cre-mediated recombination. Genomic DNA from heart samples of tamoxifen-induced cardiomyocyte-specific *Rictor* KO mice was used as positive control (pos ctrl).^85^ No template control (NTC) contains all PCR reaction components including primers but no DNA. Successful Cre-mediated recombination of the *Rictor* allele was only found in OCs and vestibular organs of HC-RicKO mice. (C) Representative images (maximum intensity projections) of the medial cochlear turn from 4-week-old mice stained with an antibody against Akt-pSer473. Myosin7a antibody, phalloidin and nuclear DAPI staining visualize the hair cells. Scale bar for all figures = 20 μm. (D) Quantification of mean fluorescence intensity (MFI) in arbitrary units (a.u.) of the Akt-pSer473 signal intensity in the medial cochlear turn of 4-week-old mice. n = 3 mice per genotype. Results are presented as means ± SDs. Student’s *t* test, ∗p < 0.05.

*Rictor* deficiency is embryonic lethal.^26^^,^^27^ In the inner ear, *Rictor* is expressed in the developing cochlea and utricle (Figure S1A) and in the adult cochlea in both IHCs and OHCs as well as supporting cells of different types (Figure S1B). In developing IHCs (Figure S1C) and OHCs (Figure S1D), *Rictor* expression increases during postnatal development. To circumvent embryonic lethality, avoid developmental effects, and investigate mTORC2 function specifically in HCs, we knocked out *Rictor* specifically in HCs in postnatal mice (HC-RicKO mice). To generate HC-RicKO mice, we crossed *Myo15*-*Cre*^+/−^ mice^28^ with *Rictor*^fl/fl^ mice.^29^ *Myo15*-*Cre* leads to Cre-mediated recombination specifically in HCs, starting at E19 in the vestibule and at P0 in the cochlear base, reaching the cochlear apex at P4 in a basal to apical gradient.^28^ At this postnatal day 4, cochlear HCs and stereocilia are developed, but the HCs undergo functional maturation until hearing onset at about P12. *Rictor*^fl/fl^ mice have a *LoxP* site introduced upstream of exon 4 and downstream of exon 5 (Figure 1A). Deletion of the two exons creates a frameshift and early stop of translation of *Rictor* mRNA.^29^^,^^30^

We compared HC-RicKO mice (*Myo15*-*Cre*^+/−^, *Rictor*^fl/fl^) to littermate controls (*Rictor*^fl/fl^), unless otherwise stated. Cre-mediated recombination was confirmed by PCR in HC-RicKO mice in the organ of Corti (OC) and vestibular organs with no recombination in the spiral ganglion (SG) of the cochlea (Figure 1B). Skin samples collected from the outer ears were used to exclude nonspecific Cre activity outside the inner ear (Figure 1B). In addition, knockout was confirmed on the protein level by immunostaining. We examined Akt-pSer473 as a readout for mTORC2 activity. Akt-pSer473 was reduced in cochlear HCs of HC-RicKO mice (Figures 1C and D). HC-RicKO mice were indistinguishable from control mice in appearance and behavior. There were no major weight differences between genotypes of the same sex, although male HC-RicKO mice were heavier than their littermate controls at ages 8 and 12 weeks (Figure S2). *Myo15* is also expressed in the anterior pituitary gland in addition to HCs^31^^,^^32^ which might disrupt mTORC2 in pituitary cells of HC-RicKO mice. In our study, we focus on the inner ear.

### HC-RicKO mice show early-onset, progressive, and profound hearing loss but normal vestibular function

**Figure 2:**
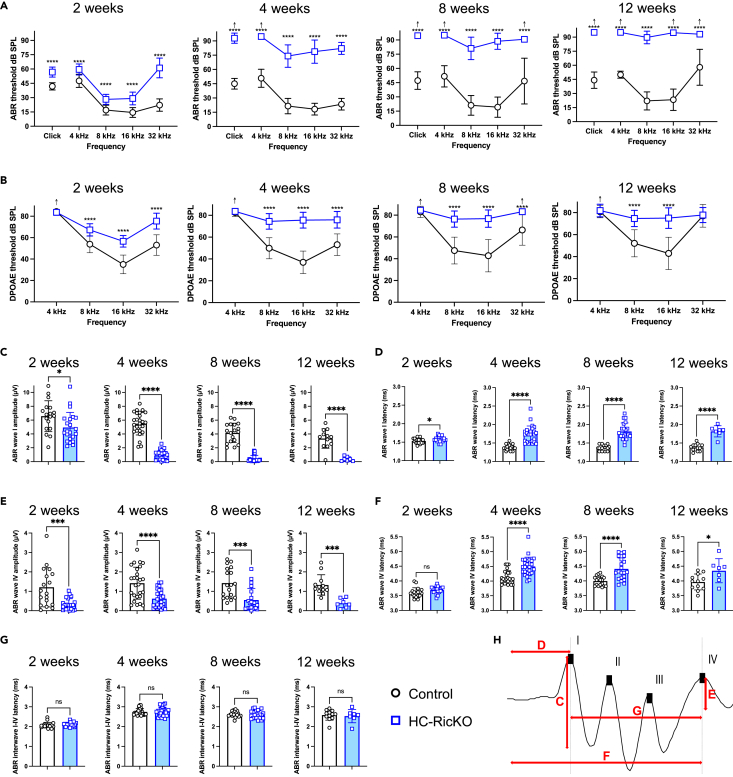
HC-RicKO mice show early-onset, progressive, and profound hearing loss (A) ABR thresholds measured for a click stimulus or frequencies indicated at 2 weeks (n = 18–22 ears from 9 to 11 animals), 4 weeks (n = 26–28 ears from 13 to 14 animals), 8 weeks (n = 18–22 ears from 9 to 11 animals), and 12 weeks of age (n = 12–16 ears from 6 to 8 animals). The arrows indicate that even at the highest SPL level tested (90 dB SPL) there was no response. Results are presented as means ± SDs. Student’s *t* test, ∗∗∗∗p < 0.0001. (B) DPOAE thresholds measured for frequencies indicated at 2–12 weeks of age in ears/animals indicated in (A). The arrows indicate that even at the highest SPL level tested (80 dB SPL) there was no response. Results are presented as means ± SDs. Student’s *t* test, ∗∗∗∗p < 0.0001. (C) ABR wave I amplitudes (peak-through) measured for an 8 kHz, 90 dB SPL stimulus in ears/animals specified in (A) at timepoints indicated. Results are presented as means ± SDs. Student’s *t* test, ∗p < 0.05, ∗∗∗∗p < 0.0001. (D) ABR wave I latencies (onset-peak) measured for an 8 kHz, 90 dB SPL stimulus in ears/animals specified in (A) at timepoints indicated. Results are presented as means ± SDs. Student’s *t* test, ∗p < 0.05, ∗∗∗∗p < 0.0001. (E) ABR wave IV amplitudes (peak-through) measured for an 8 kHz, 90 dB SPL stimulus in ears/animals specified in (A) at timepoints indicated. Results are presented as means ± SDs. Student’s *t* test, ∗∗∗p < 0.001, ∗∗∗∗p < 0.0001. (F) ABR wave IV latencies (onset-peak) measured for an 8 kHz, 90 dB SPL stimulus in ears/animals specified in (A) at timepoints indicated. Results are presented as means ± SDs. Student’s *t* test, ns not significant, ∗p < 0.05, ∗∗∗∗p < 0.0001. (G) ABR interwave I-IV latencies (peak-peak) measured for an 8 kHz, 90 dB SPL stimulus in ears/animals specified in (A) at timepoints indicated. Results are presented as means ± SDs. Student’s *t* test, ns not significant. (H) Illustrative suprathreshold ABR wave response and schematic representation of ABR waveforms analyzed in panels (C–G).

To monitor auditory function, we performed auditory brainstem response (ABR) measurements. HC-RicKO mice showed elevated hearing thresholds already at age 2 weeks, soon after hearing onset. This was strongly progressive over time, with HC-RicKO mice showing an increased hearing threshold of up to 60 dB sound pressure level (SPL) at age 4 weeks, compared with littermate controls. At age 8 weeks, ABR thresholds of HC-RicKO mice were elevated up to 69 dB SPL. At age 12 weeks, HC-RicKO mice were almost completely deaf with no measurable responses for most frequencies and a mean hearing threshold of 89.7 ± 6.7 dB SPL at 8 kHz (Figure 2A). Distortion-product otoacoustic emissions (DPOAE), which are a measure of OHC function, showed trends similar to ABRs with an increase in hearing thresholds over time in HC-RicKO mice (Figure 2B).

We then analyzed suprathreshold ABR wave amplitudes and latencies at 90 dB SPL, where responses are mostly independent of OHC amplification and therefore represent mainly IHC function.^33^ We used an 8 kHz stimulus for the analysis, where we observed measurable responses until 12 weeks of age (Figure 2A). The ABR wave I reflects electrical activity of the auditory nerve, which consists mostly of afferent nerve fibers of spiral ganglion neurons which have synaptic contacts to IHCs. ABR wave I amplitudes were significantly reduced (Figure 2C) and ABR wave I latencies were significantly increased at all time points measured in HC-RicKO mice (Figure 2D). Reduced amplitudes and increased latencies were also propagated to wave IV, which represents electrical activity in the inferior colliculus of the brainstem (Figures 2E and 2F). Most importantly, interwave I–IV latency was unchanged in HC-RicKO mice, suggesting that the auditory signal travels normally along the brainstem and that the cause of the hearing loss is in the peripheral hearing organ (Figure 2G).

While there are known sex differences in hearing with a protective role of estrogen signaling,^34^ elevations in ABR and DPOAE hearing thresholds were equally high in male and female HC-RicKO mice (Figures S3A and S3B). These results suggest that *Rictor* KO had strong effects on hearing independent of sex. *Myo15*-*Cre*^+/−^ mice had normal ABR and DPOAE hearing thresholds until 23 weeks of age. After age 36 weeks, they had elevated hearing thresholds as expected for aging, C57BL/6J mice (Figures S4A and S4B). Importantly, at age 12 weeks, ABR and DPOAE hearing thresholds in *Myo15*-*Cre*^+/−^ and *Rictor*^fl/fl^ mice were similar, excluding Cre toxicity as the cause of hearing loss (Figures S4C and S4D).

Interestingly, vestibular function was normal and indistinguishable from control mice in HC-RicKO mice at age 11–12 weeks, when HC-RicKO mice were already deaf (Table S1 and Figure S5). In aging mice, vestibular dysfunction occurs later than auditory dysfunction.^35^ Nevertheless, even at later timepoints of 28–30, 39–41, and 49–51 weeks of age, vestibular function in HC-RicKO mice was still indistinguishable from control mice (Figure S5). Therefore, *Rictor* deletion specifically in HCs leads to early-onset, progressive, and profound hearing loss but no observable vestibular dysfunction.

### Hearing loss precedes hair cell loss in HC-RicKO mice

**Figure 3:**
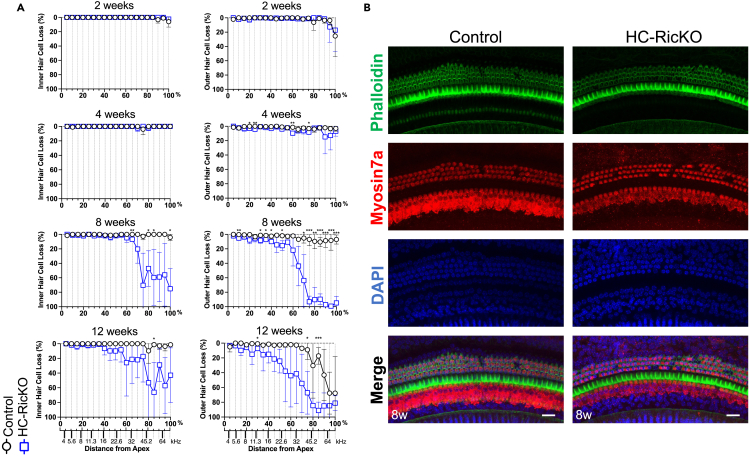
Hearing loss precedes hair cell loss in HC-RicKO mice (A) Cochleograms showing inner hair cell (left graphs) and outer hair cell (right graphs) loss in 5 percent (%) distances from the Apex at indicated timepoints. Place-frequency map calculated with formula d = (LOG10((f+6.664)/9.8)/LOG10(10))/0.0092 (where d is the distance from the Apex in % and f the frequency in kHz).^86^^,^^87^^,^^88^ n = 3–4 mice (2 weeks), 4 mice (4 weeks), 3 mice (8 weeks), and 3–4 mice (12 weeks) per genotype. Results are presented as means ± SDs. Student’s *t* test, ∗p < 0.05, ∗∗p < 0.01, ∗∗∗p < 0.001. (B) Representative images (maximum intensity projections) of the medial cochlear turn from 8-week-old mice of both genotypes. Hair cells are visualized with phalloidin, a Myosin7a antibody and nuclear DAPI staining. Scale bar for all figures = 20 μm.

HC loss is a frequent hallmark of hearing loss and often considered the main cause of sensorineural hearing loss. Given the profound hearing loss in HC-RicKO mice, we investigated HC loss as the potential underlying cause. Unexpectedly, there was no IHC or OHC loss at age 2 weeks when HC-RicKO mice already show elevated hearing thresholds (Figures 3A and S6A). Notably, at age 4 weeks when there was already profound hearing loss in HC-RicKO mice (Figure 2A), there was no IHC and almost no OHC loss (Figures 3A and S6A). At 8 and 12 weeks, there was increasing IHC and OHC loss at the cochlear base in HC-RicKO mice, and also OHC loss at age 12 weeks in control mice. However, when consulting the place-frequency map, this region mainly included frequencies over 32 kHz and was outside our tested frequency range for ABR and DPOAE. In the tested frequency range (4-32 kHz), there was minor HC loss which was insufficient to explain the profound hearing loss observed in HC-RicKO mice at ages 8 and 12 weeks. For instance, a total (100%) OHC loss cannot account for more than 40–60 dB SPL ABR hearing threshold elevation, as shown by either OHC loss^36^ or OHC dysfunction.^37^ Almost all IHCs and most of the OHCs are present in the medial cochlear turn of HC-RicKO mice at ages 8 (Figure 3B) and 12 weeks (Figure S6A). Therefore, HC loss occurs after hearing loss in HC-RicKO mice and is not the underlying cause for the profound hearing defect.

Apart from HC counts, we examined whether *Rictor* expression in HCs affected total length of the neurosensory epithelium. Cochlear length along the lateral border of IHCs did not differ between control and HC-RicKO mice at all timepoints examined (Figure S6B).

### HC-RicKO mice show abnormal stereocilia but MET channel function is present

**Figure 4:**
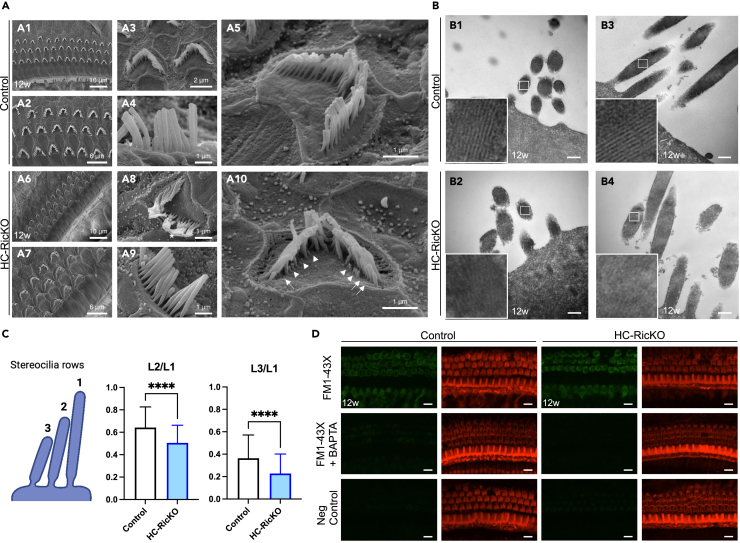
HC-RicKO mice show abnormal stereocilia but MET channel function is present (A) SEM images of control (A1-A5) and HC-RicKO (A6-A10) stereocilia. Panels A1-A2 (control) and A6-A7 (HC-RicKO) show an overview of stereocilia of different hair cell rows. Panels A3, A5 (control) and A8, A10 (HC-RicKO) show outer hair cell stereocilia. Panels A4 (Control) and A9 (HC-RicKO) show inner hair cell stereocilia. Stereocilia from outer hair cells of HC-RicKO mice show an amorphous structure on the outermost row (asterisk), shortened stereocilia (arrowheads), and missing stereocilia (arrows). All images are representative images from the medial cochlear turn of 12-week-old mice. n = 3 mice per genotype. Scale bar sizes are indicated in the corresponding figure panels. (B) TEM images of control (B1, B3) and HC-RicKO (B2, B4) stereocilia. Panels B1 (control) and B2 (HC-RicKO) show outer hair cell stereocilia. Panels B3 (control) and B4 (HC-RicKO) show inner hair cell stereocilia. Magnifications of the insets showing the paracrystalline organization of actin filaments in stereocilia are displayed inside the corresponding image panels. All images are representative images from the medial cochlear turn of 12-week-old mice. n = 3 mice per genotype. Scale bar for all figures = 200 nm. (C) Analysis of outer hair cell stereocilia length in the medial cochlear turn, where the length from the middle (L2) and small (L3) stereocilia rows was normalized to the length of stereocilia in the tall row (L1). 45 cells from 3 mice were analyzed per genotype. Results are presented as means ± SDs. Student’s *t* test, ∗∗∗∗p < 0.0001. (D) FM1-43X staining of adult cochlear hair cells. Images are maximum intensity projections from the medial cochlear turn. Cochleae of 12-week-old mice were either perfused with FM1-43X (green), FM1-43X + BAPTA or HBSS only for the negative control (Neg Control). To identify the hair cells, actin filled stereocilia and cuticular plates were stained with phalloidin (red). Data from n = 3 mice for FM1-43X and 2 mice for FM1-43X + BAPTA staining. Scale bar for all figures = 10 μm.

Given that HC loss is not the underlying cause of hearing loss, we investigated further cochlear structures relevant for hearing. Since HC-RicKO mice suffer from both OHC (reduced DPOAEs) and IHC (ABR hearing threshold elevation >60 dB SPL) dysfunction, we focused on stereocilia, which are present in both cell types. Stereocilia are the sound sensors containing the MET channels necessary for hearing. In line with no major HC loss, stereocilia from 12-week-old HC-RicKO mice were present in three rows of OHCs and a single row of IHCs similar to control mice (Figures 4A1–A2 and A6–A7). Stereocilia are actin-filled apical protrusions in HCs, normally present in three rows of different height, with very tight regulation of height in the same row (Figures 4A3–A5). Interestingly, OHCs of HC-RicKO mice showed stereocilia of different heights in the same row and some missing stereocilia (Figures 4A10 and S7A7–A10). Notably, stereocilia from the middle and small row were significantly shortened in HC-RicKO mice (Figures 4C and S7C), while there was no difference between genotypes in the length of stereocilia from the tallest row (data not shown). In addition, some stereocilia of OHCs from HC-RicKO mice showed an amorphous structure overlaying the tallest and outermost row of stereocilia (Figures 4A8, S7A6, andA8). A similar amorphous structure is transiently seen in wildtype mice at early postnatal ages but absent in stereocilin knockout mice and might represent remnants of the tectorial membrane (TM), which is an acellular roof plate covering the HCs.^38^ The tallest and outermost row of OHC stereocilia inserts into the TM. These insertions leave imprints on the lower surface of the TM, which are visible via scanning electron microscopy (SEM). The TM of HC-RicKO mice shows visible imprints similar to control mice (Figure S7B), suggesting the outermost OHC stereocilia row was normally contacting the TM. At age 2 weeks, HC-RicKO mice showed normal stereocilia architecture, similar to control mice (Figure S8), indicating that stereocilia developed normally.

To further investigate aberrations in stereocilia and HCs, we performed transmission electron microscopy (TEM) in 12-week-old mice. We did not find any further structural defects in stereocilia in TEM examinations. OHC (Figures 4B1–B2) and IHC (Figures 4B3–B4) stereocilia showed similar thickness and appearance in HC-RicKO mice as in control mice. The paracrystalline organization of actin filaments was also similar in stereocilia of both genotypes. Lastly, the morphology of HCs was also comparable between HC-RicKO and control mice (Figures S7D1–D6).

At the apical end of the middle and small row stereocilia lie the MET channels which are connected via tip-links to the adjacent, taller stereocilium. Any deflection toward the taller stereocilium opens the MET channel which is essential for hearing function. Loss of tip-links and consequently loss of MET channel function leads to middle and small row stereocilia shortening.^28^^,^^39^ We probed MET channel functionality by staining cochleae of 12-week-old mice with the styryl dye FM1-43X, which is known to enter and stain the HCs via MET channel.^40^^,^^41^ However, uptake via endocytosis has also been reported.^42^^,^^43^ IHCs and OHCs from HC-RicKO mice were equally stained with FM1-43X similar to control mice (Figure 4D). BAPTA treatment is known to disrupt the tip-links and therefore MET channel function. BAPTA abolished FM1-43X loading of HCs, thus confirming uptake via the MET channel (Figure 4D). In the apical cochlear turn, HCs from both genotypes were similarly stained with FM1-43X (Figure S9), although weaker than in the medial cochlear turn (Figure 4D) as previously described.^40^ To summarize, HC-RicKO mice display shortened and missing stereocilia but MET channel function is present, suggesting that mTORC2 is involved in stereocilia maintenance.

### HC-RicKO mice show reduced synapse numbers

**Figure 5:**
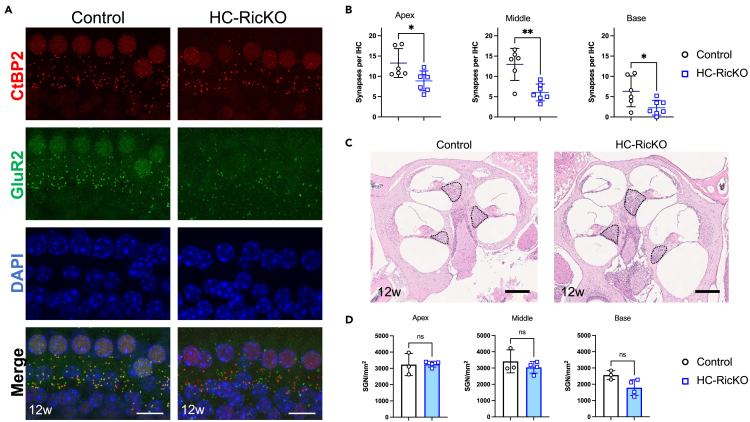
HC-RicKO mice show reduced synapse numbers (A) Representative images (maximum intensity projections) of ribbon synapses (CtBP2) and glutamate receptors (GluR2) of 12-week-old mice from inner hair cells of the medial cochlear turn. Scale bar for all figures = 10 μm. (B) Quantification of synapse counts (juxtaposed CtBP2-GluR2) per inner hair cell (IHC) in 12-week-old mice. Data from n = 6–7 ears from 4 to 5 animals. Results are presented as means ± SDs. Student’s *t* test, ∗p < 0.05, ∗∗p < 0.01. (C) Mid-modiolar cochlear sections stained with H&E from 12-week-old mice. The Rosenthal’s canal containing the spiral ganglion neurons (SGNs) is outlined with a dashed line. Scale bar for all figures = 250 μm. (D) Quantification of SGN counts in H&E stained histological sections of 12-week-old mice. Data from n = 3–4 mice. Counts were normalized to area (SGN/mm^2^) and averaged for each cochlear turn of the same ear before analysis. Results are presented as means ± SDs. Student’s *t* test, ns not significant.

Several lines of evidence link mTORC2 to synaptic function.^44^^,^^45^^,^^46^^,^^47^^,^^48^ We therefore also investigated synapse numbers and spiral ganglion neuron counts in HC-RicKO and control mice. In IHCs, synapses consist of presynaptic ribbon proteins (*Ctbp2*) and postsynaptic AMPA-type glutamate receptors (GluR).^49^ HC-RicKO mice had fewer synapses at age 12 weeks (Figure 5A). Synapses were defined and quantified as juxtaposed CtBP2 and GluR2 immunoreactive puncta. The number of synapses was significantly reduced in all cochlear turns, with the biggest reduction in the medial cochlear turn (Figure 5B). While the number of CtBP2 puncta was reduced in HC-RicKO mice, there was also a significant increase in orphan ribbons (CtBP2 puncta not juxtaposed to GluR2, Figure S10A). Of note, synaptic counts were already reduced at age 4 weeks, indicating that this is an early effect (Figure S10B). The spiral ganglion neurons (SGNs) are the neurons that innervate the IHCs and form the fibers of the cochlear nerve up to the cochlear nuclei in the brainstem. There was no significant difference in SGN counts between HC-RicKO and control mice at age 12 weeks (Figures 5C and 5D) or earlier timepoints (2, 4, or 8 weeks of age, Figures S10C–S10H). SGN loss is frequently observed as a result of HC loss. SGN counts, although not significantly, were reduced in the basal cochlear turn of HC-RicKO mice at age 12 weeks (Figure 5D). However, at this timepoint many IHCs and OHCs were already lost at the base. Hence, HC-RicKO mice show cochlear synaptopathy with loss of synapses but without SGN degeneration.

### HC-RicKO mice have a reduced synaptic F-actin network in inner hair cells

IHC synapses are crucial for hearing since they relay all auditory information to the brain. Sound-induced deflection of IHC stereocilia leads to IHC depolarization and Ca^2+^ influx at synaptic active zones. Neurotransmitter-loaded vesicles that are tethered to a synaptic ribbon protein can then fuse with the presynaptic membrane, a process dependent on the Ca^2+^ sensor otoferlin in IHCs.^50^

The first described function for mTORC2 was regulation of the actin cytoskeleton.^10^^,^^11^^,^^12^ *Rictor* knockout in different tissues was shown to regulate actin organization.^45^^,^^51^ Notably, it was also shown that a synaptic F-actin network regulates exocytosis at IHC synapses.^52^^,^^53^ Disruption of the actin cytoskeleton facilitated exocytosis^52^^,^^53^ and increased the distance between ribbon synapses and Cav1.3 channels at synaptic active zones.^52^ Given these findings and the reduced synapse number in HC-RicKO mice, we investigated synaptic active zones containing the ribbon protein (CtBP2), Cav1.3 channels, and the associated actin cytoskeleton.

**Figure 6:**
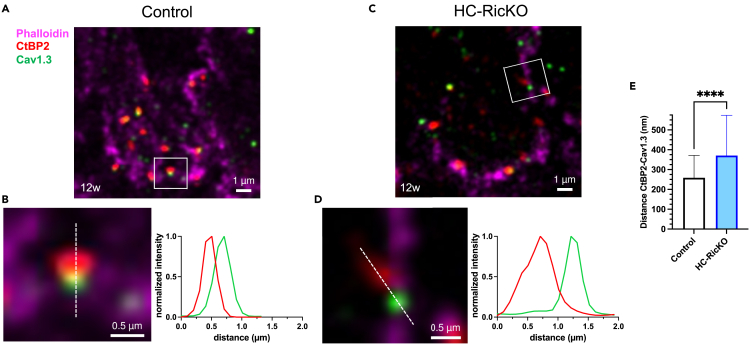
HC-RicKO mice show a reduced synaptic F-actin network in inner hair cells (A) Representative image (maximum intensity projection, 7 z-layers of 0.125 μm each) of an inner hair cell from the medial cochlear turn of a 12-week-old control mouse. Phalloidin labeled F-actin (magenta) forms a network surrounding ribbon synapses (CtBP2) and calcium channels (Cav1.3). Magnification of the inset is displayed in panel (B). Scale bar size = 1 μm. (B) Magnification from the square shown in panel (A). The ribbon synapse (CtBP2, red) lies in proximity of the calcium channel (Cav1.3, green) in control mice. The graph represents the fluorescent intensity profile of the synapse measured along the white dashed line. Scale bar size = 0.5 μm. (C) Representative image (maximum intensity projection, 7 z-layers of 0.125 μm each) of an inner hair cell from the medial cochlear turn of a 12-week-old HC-RicKO mouse. Phalloidin labeled F-actin (magenta) shows a cortical, submembranous localization. Magnification of the inset is displayed in panel (D). Scale bar size = 1 μm. (D) Magnification from the square shown in panel (C). There is a larger distance between the ribbon synapse (CtBP2, red) and calcium channel (Cav1.3, green) in HC-RicKO mice. The graph represents the fluorescent intensity profile of the synapse measured along the white dashed line. Scale bar size = 0.5 μm. (E) Quantification of the center mass distance between ribbon (CtBP2) and Cav1.3. Data from n = 4–5 ears from 2 to 3 animals aged 12 weeks, where 255 active zones per genotype were analyzed. Results are presented as means + SDs. Student’s *t* test, ∗∗∗∗p < 0.0001.

IHCs from 12-week-old control mice show a dense actin cytoskeleton surrounding the ribbon synapses (Figure 6A). After depolarization of IHCs, Ca^2+^ influx via Cav1.3 (also called alpha 1D) channels is necessary for fusion of vesicles loaded on synaptic ribbons with the presynaptic membrane.^54^ Therefore, Cav1.3 channels are closely localized to synaptic ribbons in IHCs^55^ as observed in control samples (Figure 6B). In contrast, HC-RicKO mice showed only a submembranous, cortical actin cytoskeleton (Figure 6C). In addition, the distance between Cav1.3 channels and ribbon synapses was increased in HC-RicKO mice (Figure 6D), as shown by quantification of 255 active zones per genotype (Figure 6E). Notably, a similar phenotype with a reduced synaptic actin network and increased CtBP2-Cav1.3 distance was already observed at age 4 weeks in HC-RicKO mice, again indicating that this is an early effect (Figures S11A–S11E). To summarize, HC-RicKO mice have a reduced F-actin network at synaptic active zones, affecting the spatial organization between Cav1.3 channels and synaptic ribbons.

## Discussion

Here, we show that mTORC2 is an important regulator of cochlear IHC and OHC function. HC-RicKO mice show early-onset, progressive, and profound hearing loss. Increased DPOAE thresholds in HC-RicKO mice indicate that OHC function is affected. Importantly, HCs are lost, but this is a late event occurring after deafness. Early events that may account for the hearing loss, included abnormal stereocilia and reduced synapse numbers, disrupted synaptic actin cytoskeleton and increased distances between Cav1.3 channels and ribbon synapses.

Our results are in line with a recent study showing that *Atoh1-Cre* driven deletion of *Rictor* in the entire neurosensory epithelium causes progressive and profound hearing loss.^24^ However, the authors of this previous study attributed the hearing loss to HC loss as a consequence of defective Akt signaling.^24^ In contrast, we found that hearing loss precedes HC loss in our *Rictor* knockout mice. Although the reason for this difference is not clear, one possibility is the use of different *Cre* drivers. *Atoh1*-*Cre* mediates deletion in supporting cells and HCs,^56^ whereas *Myo15*-*Cre* used in our study mediates deletion specifically in HCs.^28^ In addition, we previously reported that *Akt1* and *Akt2*/*Akt3* KO mice show hearing loss without HC loss.^25^ We also note that hearing loss precedes HC loss in many genetic mouse models.^57^^,^^58^^,^^59^^,^^60^ Similarly, in aged mice, a reduction in cochlear HC function precedes HC loss.^61^

We found abnormal stereocilia in HC-RicKO mice, in particular irregularly shortened and missing small and middle row stereocilia. This phenotype resembles that caused by mutations in actin genes *Actb* and *Actg1*.^62^^,^^63^ In adult stereocilia, the actin core is stable and actin turnover occurs at the tips,^64^^,^^65^^,^^66^ a process which is dependent on the MET current.^39^ Mechanotransduction also shapes stereocilia during development.^67^^,^^68^^,^^69^ However, HC-RicKO mice have normal stereocilia development and MET channel function is present as assessed with styryl dye staining. These results indicate that stereocilia shortening in HC-RicKO mice is not due to MET channel loss and that the MET machinery is functional even after stereocilia shortening. Similarly, stereocilia shortening after MET blockade does not lead to tip-link loss, indicating that tip-links reform or slide down during shortening.^39^ Knock-down of *Rictor* in mammalian cell lines leads to a disorganized actin cytoskeleton^11^ and reduced cell spreading.^12^ Furthermore, the F-actin/G-actin ratio is reduced in tissues of *Rictor* KO mice.^45^^,^^51^ The parallel actin filaments at the core of stereocilia and the stereocilia diameter, as visualized by TEM, appeared normal in HC-RicKO mice. mTORC2 disruption might affect actin cytoskeletal dynamics and turnover rather than stable actin structures.

The actin cytoskeleton that surrounds the ribbon synapses in IHCs was also diminished in HC-RicKO mice. As shown previously, a filamentous actin network populates the basolateral end of IHCs forming a network that surrounds the synapses.^52^^,^^53^ Disruption of this synaptic actin cytoskeleton facilitates exocytosis at the ribbon synapse and confers mechanosensitivity to Cav1.3 channels.^52^^,^^53^ Morphologically, this is mediated by increased distances between Cav1.3 channels and ribbon synapses upon disruption of the actin cytoskeleton.^52^ Similarly, HC-RicKO mice had increased distances between Cav1.3 channels and ribbon synapses at IHC active zones. The precise mechanism of how the actin cytoskeleton regulates exocytosis remains unclear. However, a mechanism where the actin cytoskeleton regulates the replenishment of vesicles at the IHC synapse has been proposed.^52^^,^^53^ Increased recruitment of vesicles may lead to exhaustion of the vesicle pool after actin cytoskeleton disruption,^53^ affecting exocytotic function in the long term. While the functional implications of these effects, measured *in vitro,* on hearing function are difficult to predict, it is tempting to speculate that the disrupted synaptic actin cytoskeleton and increased Cav1.3-ribbon distance in HC-RicKO mice are contributing to hearing loss. It may lead to disrupted synaptic function and the observed reduction in synaptic numbers. Similarly, it was recently shown that disrupting IHC exocytosis leads to loss of ribbon synapses and subsequently to IHC death.^70^

In contrast to hearing loss and impaired auditory HC function, we did not observe an obvious vestibular phenotype in HC-RicKO mice. Why is cochlear, but not vestibular HC function affected in HC-RicKO mice? Although cochlear and vestibular HCs share many morphological and molecular features, there are also several important differences. Vestibular HCs retain the kinocilium, a structure which is only transiently present in cochlear HCs, and have a different composition in basolateral K^+^ channels which repolarize HCs.^71^ Another considerable difference is found in synaptic specializations between the two sensory systems, although they share common molecular components. While in IHCs a single ribbon contacts an afferent fiber in a bouton synapse, vestibular HCs contain calyceal synapses apart from bouton and dimorphic (bouton and calyx) synapses, where multiple ribbons contact a large afferent terminal.^71^ In addition, single afferent fibers contact multiple vestibular HCs, whereas cochlear afferent fibers exquisitely innervate one IHC.^71^ Notably, vestibular HCs have a different organization of Cav1.3 channels at the ribbon synapse^72^ and even show Ca^2+^ independent non-quantal transmission.^71^ Similarly to HC-RicKO mice, Cav1.3 KO mice are deaf with no vestibular dysfunction, despite greatly reduced Ca^2+^ influx.^54^^,^^73^^,^^74^ This might be due to normal non-quantal synaptic transmission in Cav1.3 KO mice, which depends on potassium currents that are unaffected in vestibular HCs of Cav1.3 KO mice.^74^ Therefore, a synaptic phenotype might not be apparent in vestibular HCs upon loss of *Rictor*. Another property found only in the hearing organ of mammals is the somatic electromotility of OHCs.^75^ We found reduced DPOAEs in HC-RicKO mice indicating that OHC function is disrupted. While this might be explained in part by the abnormal stereocilia, it is possible that a cytoskeletal defect also affects OHC electromotility and amplification function. OHCs have specialized structures in their lateral wall important for electromotility, including a cortical lattice cytoskeletal complex containing actin and spectrin filaments.^76^^,^^77^ It will be interesting to investigate whether potential cortical lattice defects impact OHC electromotility in HC-RicKO mice.

What are the effectors of mTORC2 signaling in HCs? mTORC2 regulates the actin cytoskeleton via its substrate PKC alpha, but also mechanisms involving Rho GTPases have been described.^11^^,^^12^^,^^13^^,^^14^ PKC alpha may be involved in the regulation of synaptic processes in IHCs^16^ and in regulating OHC electromotility.^17^ Deletion of *RhoA, Rac1* and *Cdc42* all lead to abnormal stereocilia^78^^,^^79^^,^^80^^,^^81^ and RhoA, Rac1, and Cdc42 small Rho GTPases have been proposed to regulate OHC motility.^82^ Alternatively, mTORC2 might regulate the actin cytoskeleton via Girdin,^83^ which is an Akt substrate and actin binding protein.^84^

Our results reveal mTORC2 as a critical regulator of auditory HC function by regulating the actin cytoskeleton. mTORC2 is indispensable for both IHC and OHC function, regulating different structures such as stereocilia and synapses. Therefore, mTORC2 is a central regulator of cochlear HCs and thus hearing. It will be of interest to further determine mTORC2 regulated processes in HCs, as well as the precise underlying molecular mechanisms. Of note, *Rictor* expression decreases in both IHCs and OHCs with aging concomitant with a decrease in HC function.^61^ Therefore, it will also be interesting to determine the role of mTORC2 in aging HCs, as this might open new perspectives to treat sensorineural hearing loss.

### Limitations of the study

In this study, we show that mTORC2 is involved in stereocilia and synapse maintenance of auditory HCs and that mTORC2 regulates auditory HC function. However, how mTORC2 signals to these structures mechanistically remains elusive. We did not see any vestibular phenotype in HC-RicKO mice using behavioral assessment. More sensitive methods might reveal some vestibular defects in HC-RicKO mice, and the role of mTORC2 in vestibular HCs needs to be addressed more precisely in future studies.

## STAR★Methods

### Key resources table

**Table:** 

REAGENT or RESOURCE	SOURCE	IDENTIFIER
**Antibodies**		

Goat Anti-Mouse IgG Fab Fragment	Jackson ImmunoResearch	Cat#115-007-003; RRID:AB_2338476
Mouse monoclonal anti-myosin7a IgG1	Developmental Studies Hybridoma Bank	Cat#138-1s; RRID:AB_2282417
Mouse monoclonal anti-CtBP2 IgG1	BD Biosciences	Cat#612044; RRID:AB_399431
Mouse monoclonal anti-GluR2 IgG2a	Sigma Aldrich Chemie GmbH	Cat#MAB397; RRID:AB_2113875
Rabbit monoclonal anti-phospho-Akt (Ser473) IgG	Cell Signaling Technology	Cat#4060; RRID:AB_2315049
Rabbit polyclonal anti-Cav1.3 IgG	Alomone Labs	Cat#ACC-005; RRID:AB_2039775
Alexa Fluor 488 goat anti-rabbit IgG	Thermo Fisher Scientific	Cat#A11008; RRID:AB_143165
Alexa Fluor 568 goat anti-mouse IgG	Thermo Fisher Scientific	Cat#A11031; RRID:AB_144696
Alexa Fluor 488 goat anti-mouse IgG2a	Thermo Fisher Scientific	Cat#A21131; RRID:AB_2535771
Alexa Fluor 647 goat anti-mouse IgG1	Thermo Fisher Scientific	Cat#A21240; RRID:AB_2535809
Alexa Fluor 568 goat anti-mouse IgG1	Thermo Fisher Scientific	Cat#A21124; RRID:AB_2535766

**Chemicals, peptides, and recombinant proteins**		

Ketamin	Streuli Tiergesundheit AG	Ketanarkon® 100 ad us. Vet.
Xylazin	Streuli Tiergesundheit AG	Xylazin Streuli ad us. vet.
Acepromazine	Fatro S.p.A.	Prequillan ad us. vet.
Pentobarbital	Streuli Tiergesundheit AG	Esconarkon® ad us. vet.
Phosphate Buffered Saline (PBS)	Thermo Fisher Scientific	Cat#20012019
4% buffered formaldehyde	Biosystems Switzerland AG	Cat#84-1951-01
Methanol	Sigma Aldrich Chemie GmbH	Cat# 1.06035; CAS: 67-56-1
MolDECAL 10	Biosystems Switzerland AG	Cat#51413
Triton X-100	Sigma Aldrich Chemie GmbH	Cat#T9284
Bovine Serum Albumin	Reactolab	Cat#SP-5050
Goat Serum	Thermo Fisher Scientific	Cat#16210064
Alexa Fluor 568 Phalloidin	Thermo Fisher Scientific	Cat#A12380
ActinGreen 488 ReadyProbes Reagent	Thermo Fisher Scientific	Cat#R37110
DAPI	Sigma Aldrich Chemie GmbH	Cat#D9542
ProLong Gold Antifade Mountant	Thermo Fisher Scientific	Cat#P36930
Vectashield Mounting Medium	Reactolab	Cat#H-1000
Vectashield Plus Mounting Medium	Reactolab	Cat#H-1900
Hanks’ Balanced Salt Solution with calcium	Thermo Fisher Scientific	Cat#14025092
HEPES buffer	Thermo Fisher Scientific	Cat#15630
FM1-43FX	Thermo Fisher Scientific	Cat#F35355
1,2-Bis(2-Aminophenoxy)ethane-N,N,N′,N′-tetraacetic acid (BAPTA)	Sigma Aldrich Chemie GmbH	Cat#A4926
Hanks’ Balanced Salt Solution without calcium	Thermo Fisher Scientific	Cat#14175095
4% paraformaldehyde	Invitrogen AG	Cat#FB002
Fetal bovine serum	Thermo Fisher Scientific	Cat#10082147
25% Glutardialdehyde	Carl Roth	Cat#4157.2
Leibovitz’s L-15 solution	Thermo Fisher Scientific	Cat#21083
25% Glutardialdehyde	Electron Microscopy Science	Cat#16300
16% Formaldehyde	Electron Microscopy Science	Cat#15700

**Experimental models: Organisms/strains**		

Mouse: *Myo15*-*Cre*^+/−^: Myo15a^tm1.1(cre)Ugds^: C57BL/6J	Caberlotto et al.^28^	N/A
Mouse: *Rictor*^fl/fl^: Rictor^tm1Rueg^: C57BL/6J	Bentzinger et al.^29^	N/A

**Oligonucleotides**		

5′-AGGGACCTGACTCCACTTTGGG-3′	Caberlotto et al.^28^	Myo15-Forward
5′-GGAACTGACCTTTCTTAGAGATCTTGGG-3′	Caberlotto et al.^28^	Myo15-Reverse
5′-TGGTGCACAGTCAGCAGGTTGG-3′	Caberlotto et al.^28^	Cre-Reverse
5′-TTATTAACTGTGTGTGGGTTG-3′	Bentzinger et al.^29^	Forward-Rictor-fl
5′-CGTCTTAGTGTTGCTGTCTAG-3′	Bentzinger et al.^29^	Reverse-Rictor-fl
5′-CAGATTCAAGCATGTCCTAAGC-3′	Bentzinger et al.^29^	Reverse-Exc-Rictor-fl

**Software and algorithms**		

Fiji (Fiji is just ImageJ)	Schindelin et al.^89^	https://imagej.net/software/fiji/; RRID:SCR_002285
Nikon NIS software	Nikon AG Instruments	https://www.microscope.healthcare.nikon.com; RRID:SCR_014329
Imaris 9.9.0 software	Bitplane, Oxford Instruments	https://imaris.oxinst.com; RRID:SCR_007370
QuPath version 0.3.0	Bankhead et al.^91^	https://qupath.github.io; RRID:SCR_018257
Huygens Professional version 21.10	Scientific Volume Imaging	https://svi.nl/Huygens-Professional; RRID:SCR_014237
Prism 9 software	GraphPad software	https://www.graphpad.com; RRID:SCR_002798

### Resource availability

#### Lead contact

Further information and requests for resources and reagents should be directed to and will be fulfilled by the lead contact, Daniel Bodmer (Daniel.Bodmer@usb.ch).

#### Materials availability

This study did not generate new unique reagents.

### Experimental model and subject details

#### Animal handling and mice

All animal experiments were approved by and carried out according to regulations of the Animal Care Committee of the Canton of Basel, Switzerland in accordance with the Animal Welfare Act and the Animal Protection Ordinance of Switzerland. All mice were bred and maintained in the animal facility of the Department of Biomedicine, University of Basel, Switzerland. Mice were housed at specific pathogen free (SPF) conditions in temperature-controlled rooms under a 12 h light cycle with access to food and water *ad libitum*.

HC-RicKO mice were generated by crossing *Myo15*-*Cre*^+/−^ mice^28^ with *Rictor*^fl/fl^ mice^29^ and maintained on a C57BL/6JRj background. Mice of either sex were randomly assigned to experimental groups at ages indicated in the figures and figure legends.

### Method details

#### Genotyping

PCR genotyping was performed as previously described for the *Myo15*-*Cre*^28^ and the *Rictor*^fl/fl^ mice.^29^ Briefly, the Myo15-Forward (F) primer (5′-AGGGACCTGACTCCACTTTGGG-3′) and the Myo15-Reverse (R) primer (5′-GGAACTGACCTTTCTTAGAGATCTTGGG-3′) were used to detect the wild-type allele (587 bp) and the Myo15-F with the cre-R primer (5′-TGGTGCACAGTCAGCAGGTTGG-3′) were used to detect the *Myo15*-*Cre* allele (500 bp). The F-Rictor-fl (5′- TTATTAACTGTGTGTGGGTTG-3′) and the R-Rictor-fl (5′-CGTCTTAGTGTTGCTGTCTAG-3′) primers were used to detect floxed (295 bp) or wild-type *Rictor* allele (197 bp). F-Rictor-fl primer and R-Exc-Rictor-fl primer (5′-CAGATTCAAGCATGTCCTAAGC-3′) were used to amplify a PCR product of 280 bp and confirm the presence of the recombined *Rictor* allele. No PCR product was detectable in wild-type or unrecombined *Rictor* allele (Figure 1). Each experimental mouse was genotyped at least twice, once at neonatal ages and once confirmed at adult ages after hearing measurements and/or tissue collection.

#### Hearing measurements

Hearing function was assessed by measuring auditory brainstem responses (ABR) and distortion-product otoacoustic emissions (DPOAE) using an RZ6-A-P1 Multi-I/O processor from Tucker-Davis Technologies (TDT, Alachua, FL, USA). The speakers were calibrated with a 1/4″ free-field 377C01 microphone and 426B03 preamplifier (PCB Piezotronics, Depew, NY, USA) using BioSigRZ and RPvdsEx software (TDT, Alachua, FL, USA).

The mice were anesthetized using an intraperitoneal injection of ketamine (80 mg/kg), xylazine (12 mg/kg), and acepromazine (2 mg/kg). Body temperature was maintained at 37°C and monitored with a rectal probe. Ophthalmic ointment was applied following anesthesia induction to prevent corneal damage.

ABRs were measured using an RZ6-A-P1 processor with RA4PA preamplifier, RA4LI headstage, and an MF1 speaker in a closed-field setup using the BioSigRZ software (all from TDT, Alachua, FL, USA) and subcutaneous needle electrodes (inserted at the vertex, ipsilateral ear and a grounding electrode at the hind hip) placed in a sound attenuating chamber with a built in Faraday cage. Stimuli were presented either as clicks (duration 0.1 ms, repetition rate 21/s) or tone bursts (gate time 0.2 ms, duration 2.5 ms, repetition rate 21/s) at 4, 8, 16, and 32 kHz. Hearing thresholds were investigated by reducing the sound intensity in 5 dB steps from 90 dB SPL. The hearing threshold was defined as the lowest intensity in dB SPL to generate a visually detectable first and second peak. Acquisition was performed using 100 Hz highpass, 3 kHz lowpass and 50 Hz notch filters, and responses were averaged 512 times. ABR responses were collected and saved offline for later analysis. ABR suprathreshold waves at 8 kHz 90 dB SPL were analyzed for amplitudes and latencies.

DPOAEs were measured using an RZ6-A-P1 processor, two MF1 speakers in a closed-field setup (TDT, Alachua, FL, USA), and a ER10B+ microphone with preamplifier (Etymotic Research, Elk Grove Village, IL, USA). Two continuous primary tones were presented (f1 and f2, each from a separate speaker) with f2/f1 = 1.2 and geometrically centered at test frequencies of 4, 8, 16, and 32 kHz. The levels of the two primary tones were kept equal and decremented for each test frequency from 80 dB SPL to 20 dB SPL in 5 dB steps. Data was collected every 20.971 ms and averaged 128 times. The DPOAE threshold was defined as the lowest intensity in dB SPL to generate a detectable DPOAE at the frequency 2f1-f2.

#### Vestibular phenotyping

Assessment of vestibular function was performed by using a behavioral vestibular phenotyping pipeline as described in.^89^ Briefly, a score was calculated by observing behavioral signs (head tossing or circling behavior), and by performing a trunk curl test, a contact righting test, and a swim test. Scores were either no/normal (0) or yes/deficient (1) for the behavioral signs and trunk curl test, respectively; or scored from 0 to 3 in the contact righting and swimming test as shown in Table S1.

#### Immunofluorescence staining

Mice were euthanized using an intraperitoneal injection of 150 mg/kg pentobarbital (Streuli, Uznach, Switzerland), perfused transcardially with 1x PBS (20012019, Thermo Fisher Scientific, Reinach, Switzerland) and the inner ear was immediately isolated from the temporal bone and placed in fixative. All inner ears were fixed overnight in 4% buffered formaldehyde (84-1951-01, Biosystems, Muttenz, Switzerland) except for ears which were stained with an anti-Cav1.3 antibody, which were quickly perfused with and fixed for 30 min in 100% methanol at −20°C according to Vincent et al.^52^ After fixation, inner ears were washed and decalcified using a 10% EDTA solution (MolDECAL 10, 51413, Biosystems, Muttenz, Switzerland) at 37°C for 4 to 48 h depending on the age of the mouse. After decalcification, small holes were made in the cochlear capsule, the round and oval windows as well as a small opening on the apex for easier penetration of buffers. Inner ear samples were washed and permeabilized for 2 h at room temperature using a 5% Triton X-100 (T9284, Sigma Aldrich Chemie GmbH, Steinheim, Germany) solution (except for anti-Cav1.3 staining where no permeabilization was performed). Blocking was performed using a 5% Bovine Serum Albumin (BSA, SP-5050, Reactolab, Servion, Switzerland), 5% Goat Serum (16210064, Thermo Fisher Scientific, Reinach, Switzerland), and T-PBS (0.1–0.2% Triton X-100 in 1x PBS) solution for 2 h at room temperature. For primary antibodies that were raised in mice, a Goat Anti-Mouse IgG Fab Fragment was added to the blocking solution (115-007-003, Jackson ImmunoResearch, St. Thomas' Place, UK) and a short post-fixation in 4% buffered formaldehyde was applied (except for anti-Cav1.3 staining where no formaldehyde was used). Primary antibodies were diluted in 1% BSA, 3% Goat serum, T-PBS and incubated for 26-28 h at room temperature. The following primary antibodies and dilutions were used: Myosin7a (1:100, mouse IgG1, 138-1s, developed by Orten, D.J., Boys Town National Research Hospital, obtained from the Developmental Studies Hybridoma Bank, created by the NICHD of the NIH and maintained at The University of Iowa, Department of Biology, Iowa City, IA 52242), CtBP2 (1:200, mouse IgG1, 612044, BD Biosciences, Allschwil, Switzerland), GluR2 (1:1000, mouse IgG2a, MAB397 clone 6C4 Chemicon, Sigma Aldrich Chemie GmbH, Steinheim, Germany), *p*-Akt S473 (1:200, 4060 CST, BioConcept, Allschwil, Switzerland), and Cav1.3 (1:200, ACC-005, Alomone Labs, Jerusalem, Israel). After primary antibody incubation, inner ears were washed and stained with appropriate secondary antibodies overnight at 4°C diluted 1:500 in 1% BSA, 3% Goat serum, and T-PBS. The following secondary antibodies were used (all from Thermo Fisher Scientific, Reinach, Switzerland): Alexa Fluor 488 goat anti-rabbit IgG (A11008), Alexa Fluor 568 goat anti-mouse IgG (A11031), Alexa Fluor 488 goat anti-mouse IgG2a (A21131), Alexa Fluor 647 goat anti-mouse IgG1 (A21240), Alexa Fluor 568 goat anti-mouse IgG1 (A21124). Phalloidin was added to the secondary antibody solution, either Alexa Fluor 568 conjugated (1:1000, A12380) or Alexa Fluor 488 conjugated (1 drop in 2mL, R37110, both from Thermo Fisher Scientific, Reinach, Switzerland). After secondary antibody incubation, inner ears were washed and incubated with DAPI 1 μg/ml (D9542, Sigma Aldrich Chemie GmbH, Steinheim, Germany) for 30 min at room temperature. After final washings steps, the inner ears were dissected for whole mount preparations. The cochlea was dissected into 3–4 pieces as described in.^90^ Finally, the dissected inner ears were mounted on slides either in Prolong Gold (P36930, Thermo Fisher Scientific, Reinach, Switzerland), Vectashield Mounting Medium (H-1000, Reactolab, Servion, Switzerland) or Vectashield Plus Mounting Medium (H-1900, Reactolab, Servion, Switzerland).

#### FM1-43X styryl dye staining of adult cochleae

12 week old mice were euthanized as described above but without transcardial perfusion and decapitated. The inner ears were extracted and quickly transferred to a sterile plastic Petri dish filled with ice-cold Hanks’ Balanced Salt Solution (HBSS, with calcium, 14025092, Thermo Fisher Scientific, Reinach, Switzerland) and 10mM HEPES buffer (15630, Thermo Fisher Scientific, Reinach, Switzerland). Under a stereomicroscope, the stapes was removed from the oval window, and the round and oval window membranes as well as the apex were punctured with a needle to allow HBSS flow through the cochlea. The cochleae were then perfused through the oval and round windows with ice-cold HEPES buffered HBSS for 15 min followed by 30 s perfusion with ice-cold 5μM of FM1-43FX (F35355, Thermo Fisher Scientific, Reinach, Switzerland) diluted in HEPES buffered HBSS. For all samples with 5mM BAPTA (A4926, Sigma Aldrich Chemie GmbH, Steinheim, Germany) treatment, ice-cold HEPES buffered HBSS without calcium was used in all steps (14175095, Thermo Fisher Scientific, Reinach, Switzerland). Negative control samples were perfused with ice-cold HEPES buffered HBSS without FM1-43X. Finally, cochleae were perfused with ice-cold HEPES buffered HBSS to wash out styryl dye through the oval window and washed by quickly placing in HEPES buffered HBSS. The cochleae were then fixed overnight with 4% paraformaldehyde (PFA, FB002, Invitrogen AG, Reinach, Switzerland), decalcified for 8 h as described above with 10% EDTA, permeabilized with 5% Triton X-100 and 10% fetal bovine serum (FBS, 10082147, Thermo Fisher Scientific, Reinach, Switzerland) in 1x PBS for 2 h at room temperature, stained with Phalloidin 568 (1:500, A12380, Thermo Fisher Scientific, Reinach, Switzerland) overnight at 4°C, dissected, and mounted on slides with Vectashield Plus mounting medium.

#### Image acquisition

Images were acquired either with a Nikon Eclipse Ti microscope, equipped with an A1 point-scanning confocal unit (Nikon AG Instruments, Egg, Switzerland), or with a Nikon Eclipse Ti microscope, equipped with a Yokogawa CSU-W1 spinning disk (pinhole size 25 μm, unless otherwise stated) confocal unit (Nikon AG Instruments, Egg, Switzerland), and a Photometrics Prime 95B camera (cell size: 11 μm × 11 μm).

For point-scanning confocal, either a 40× air objective (numerical aperture 0.95) or a 100× oil objective (numerical aperture 1.45) was used. Fluorescence was excited with 402, 488, 560, 647 nm lasers and emission was filtered with 450/50 (PMT detector), 525/50 (GaAsP detector), 595/50 (GaAsP detector), 700/75 (PMT detector) bandpass filters, respectively. The pinhole size was set to 1 AU. The laser intensity and detector gain (offset was set to 0) were adjusted for each channel (on control samples) to prevent over- and undersaturation of the images. The same settings were applied to all samples belonging to the same analysis group.

For the spinning disk confocal, either a 20× air objective (numerical aperture 0.75) or a 40× air objective (numerical aperture 0.95) was used. Fluorescence was excited with 405, 488, 561, 640 nm lasers and emission was filtered with 460/50, 525/50, 630/75, 700/75 bandpass filters, respectively. The laser intensity and camera exposure time were adjusted for each channel to prevent over- and under-saturation of the images. The same settings were applied to all samples belonging to the same analysis group.

For *p*-Akt staining, images were taken with the spinning disk confocal and the 40× objective as z-stacks (step size of 0.6 μm), pixel size 0.28 μm.

For HC quantification, images were taken with the spinning disk confocal and the 20× objective as z-stacks (step size of 0.6 μm), pixel size 0.55 μm. Representative images for figures were taken with the point-scanning confocal, 40× objective with an additional 1.192 zoom, as z-stacks (step size of 0.50 μm), scans were averaged 2 times per XY section, pixel size 0.26 μm.

For FM1-43X styryl dye staining, images were taken with the spinning disk confocal (pinhole size 50 μm) and the 40× objective as z-stacks (step size of 0.6 μm), pixel size 0.28 μm.

For synapse number quantification, images were taken with the point-scanning confocal, 40× objective with an additional 2.0 zoom, as z-stacks (step size of 0.45 μm), scans were averaged 4 times per XY section, pixel size 0.16 μm. Representative images for figures were taken with the point-scanning confocal, 100× objective with an additional 1.0 zoom, as z-stacks (step size of 0.15 μm), pixel size 0.12 μm.

For imaging the synaptic actin cytoskeleton, CtBP2/ribbon synapses, and Cav1.3×channels, images were taken with the point-scanning confocal, 100× objective with an additional 1.22 zoom, as z-stacks (step size of 0.125 μm), scans were averaged 4 times per XY section, pixel size 0.1 μm.

#### Histology and hematoxylin and eosin (H&E) staining

For histological analysis, inner ears were collected, fixed, and decalcified as described for immunofluorescence staining and processed using a 29 h paraffin embedding program. 7.5μm sections were cut, stained with H&E using a Gemini autostainer (Thermo Fisher Scientific, Reinach, Switzerland), and imaged using a Hamamatsu NanoZoomer S60 (Hamamatsu Photonics, Hamamatsu City, Japan) slide scanning device using a 40× magnification (numerical aperture 0.75), pixel size 0.23 μm.

#### Image analysis and quantification

For quantification of Akt-pSer473 signal intensity, Fiji (Fiji is just ImageJ)^91^ was used. Briefly, 3D stacks with equal amounts of z-layers (10 layers) were maximum intensity projected. Mean signal intensity for Akt-pSer473 staining in the HC region as well as of the background were measured. The background was subtracted from the signal of the HC region, results were normalized to control (mean = 1) and analyzed. For each cochlear turn, 3–5 regions were measured.

For HC counting and measuring cochlear length, images were maximum intensity projected, all segments from the same cochlea were stitched, and a scale bar was added using the microscope’s Nikon NIS software. Then, using Fiji^91^ and the Measure_Line plugin from Mass Eye and Ear, Eaton-Peabody Laboratories, Histology Core (https://masseyeandear.org/research/otolaryngology/eaton-peabody-laboratories/histology-core), a line was drawn along the IHC lateral margin to measure the length of the cochlear sensory epithelium (after pixel calibration using the scale bar as described on the plugin site). The same line and plugin were used to generate a mask separating the cochlea into 5% distances from the Apex which was saved as region of interest (ROI). For each 5% distance from the Apex, IHCs, OHCs, and respective missing HCs were counted to plot the results as cochleogram as described in.^92^ The place-frequency map was calculated using the formula d = (LOG10((f+6.664)/9.8)/LOG10(10))/0.0092 (where d is the distance from the Apex in percent and f the frequency in kHz) as described in^86^ and derived from.^87^^,^^88^

Stereocilia length was analyzed as described in Caberlotto et al.^28^ Briefly, the length of measurable stereocilia in scanning electron microscopy (SEM) images from the middle and small stereocilia rows was normalized to the length of stereocilia in the tall row. 6–45 cells from the apical and medial cochlear turn of 3 mice were analyzed per genotype. The observer was blinded to genotype.

Synapse numbers were quantified using Imaris 9.9.0 software (Bitplane, Oxford Instruments), where the spot detection tool was used to detect CtBP2 and GluR2 puncta in the IHC region. Synapses were defined as juxtaposed CtBP2 and GluR2 spots (<1 μm). The total amount of synapses was divided by the total number of IHCs in the imaged segment (usually around 20, identified by DAPI-stained nuclei), to display synapse counts per single IHC. 3–6 segments per cochlear turn were imaged and examined, averaged for the same ear, and analyzed for comparison between genotypes. Orphan ribbons (CtBP2 puncta not juxtaposed to GluR2) were calculated by subtracting the number of (juxtaposed) synapses from the total number of CtBP2 puncta.

SGN counts were quantified in H&E stained histological sections, on every third slide where three consecutive 7.5μm sections from serial cuts of paraffin blocks were collected along the entire cochlear diameter. Analysis was performed in QuPath version 0.3.0,^93^ where the region of the Rosenthal’s spiral canal was manually annotated and categorized into cochlear region (Apex, Middle, Base). The area, as well as the SGN numbers, in these regions were then measured in QuPath with the Cellpose QuPath extension (https://github.com/BIOP/qupath-extension-cellpose, version 0.3.5) using a custom script, where we used a livecell Cellpose 2D model – “L2”^94^ to count the SGNs using parameters: diameter (35) and pixelSize (0.3). Segmented cells were validated based on shape features: SGNs were excluded if they met criteria size (<40 μm^2^) or circularity (<0.375). The counts were normalized to area (SGN/mm^2^), averaged for each cochlear turn of the same ear, and analyzed for comparison between genotypes.

The distance between CtBP2 (ribbon) and Cav1.3 channels was measured as described previously.^52^^,^^72^ Briefly, images were deconvolved with Huygens Professional version 21.10 (Scientific Volume Imaging, The Netherlands) using the express deconvolution standard profile. Then, using the Fiji JACoP plugin,^95^ the center mass distance was measured using objects based methods. 2–3 regions in the medial cochlear turn were imaged each spanning around 13 IHCs. 255–275 active zones (CtBP2 and Cav1.3 juxtaposed patches) were measured per genotype, coming from 4 to 5 ears from 2 to 3 animals. To plot intensity profiles the command ‘plot profile’ was used in Fiji, the data was saved and normalized.

#### Electron microscopy (EM) studies

For EM studies, 2 and 12 week old mice were euthanized as described for immunofluorescence and the inner ears were quickly isolated.

For scanning electron microscopy (SEM), the inner ears were fixed in 2.5% Glutardialdehyde (4157.2, Carl Roth, Karlsruhe, Germany) overnight at 4°C, decalcified as described for immunofluorescence for 4-7h, washed, and then the cochlear capsule was carefully opened under a stereomicroscope. Afterward, the samples were dehydrated in ascending series of ethanol, critical point dried, mounted, and gold sputtered. Images were acquired using a Philips XL30 ESEM (Philips, Amsterdam, Netherlands) SEM microscope at 5-10kV.

For transmission electron microscopy (TEM), the samples were processed as described in.^96^ Briefly, inner ears were quickly transferred to Leibovitz’s L-15 solution (21083, Thermo Fisher Scientific, Reinach, Switzerland), where the round and oval windows as well as the apical portion of the cochlea were opened. The cochlea was then gently and slowly perfused with 1% glutaraldehyde (16300, all reagents for TEM are from EMS, Lucerna-Chem, Luzern, Switzerland)/4% formaldehyde (15700) in 0.15 M cacodylate buffer (pH 7.2) supplemented with 2 mM CaCl2 and left in the same fixative solution for 1h. Afterward, the samples were fixed with 2.5% glutaraldehyde in cacodylate buffer for 1h. Decalcification was performed as described for immunofluorescence for 3 days and then the cochleae were bisected longitudinally. The cochleae were shortly immersed in 2.5% glutaraldehyde in cacodylate buffer for 15 min and placed for 1h in 2.5% glutaraldehyde in cacodylate buffer with 1% tannic acid to stain the links. Post-fixation was performed in 1% osmium tetroxide/1.5% potassium ferrocyanide in cacodylate buffer for 1h at 4°C in the dark and the samples were then dehydrated on ice in ascending ethanol series. Embedding was performed in a mixture of resin and propylene oxide. Resin polymerization was carried out in an oven at 60°C for 48h. Ultrathin sections were cut with diamond knives, collected with Formvar/carbon-coated copper grids (FCF2010-CU), and impregnated with uranyl acetate and lead citrate. Images were acquired with a FEI Tecnai G2 Spirit (FEI company, Hillsboro, OR, USA) TEM microscope at 80kV and a Veleta camera (EMSIS, Münster, Germany) operated by RADIUS software from EMSIS.

### Quantification and statistical analysis

Results are presented as means ± SDs. The statistical analysis was performed and graphs were created with Prism 9 software (GraphPad software, La Jolla, CA, USA). To determine differences between two groups, a student’s unpaired t-test was used. Ranked data (balance scores) were analyzed with a Mann-Whitney test. The results were considered statistically significant with a p value <0.05. Asterisks in the figures summarize the degree of significance (∗p < 0.05; ∗∗p < 0.01; ∗∗∗p < 0.001; ∗∗∗∗p < 0.0001). Statistical details are indicated in the figure legends.

## Data Availability

• All data reported in this paper will be shared by the lead contact upon request. • This paper does not report original code. • Any additional information required to reanalyze the data reported in this paper is available from the lead contact upon request.
