# Supplementary material for: mTORC2 regulates auditory hair cell structure and function
Source: iScience. 2023 Aug 19;26(9):107687. doi: 10.1016/j.isci.2023.107687 (PMC10484995; doi:10.1016/j.isci.2023.107687)
Supplement: Document S1. Figures S1–S11 and Table S1 [file mmc1.pdf]

iScience, Volume *26*

Supplemental information

mTORC2 regulates auditory hair cell structure and function

Maurizio Cortada, Soledad Levano, Michael N. Hall, and Daniel Bodmer

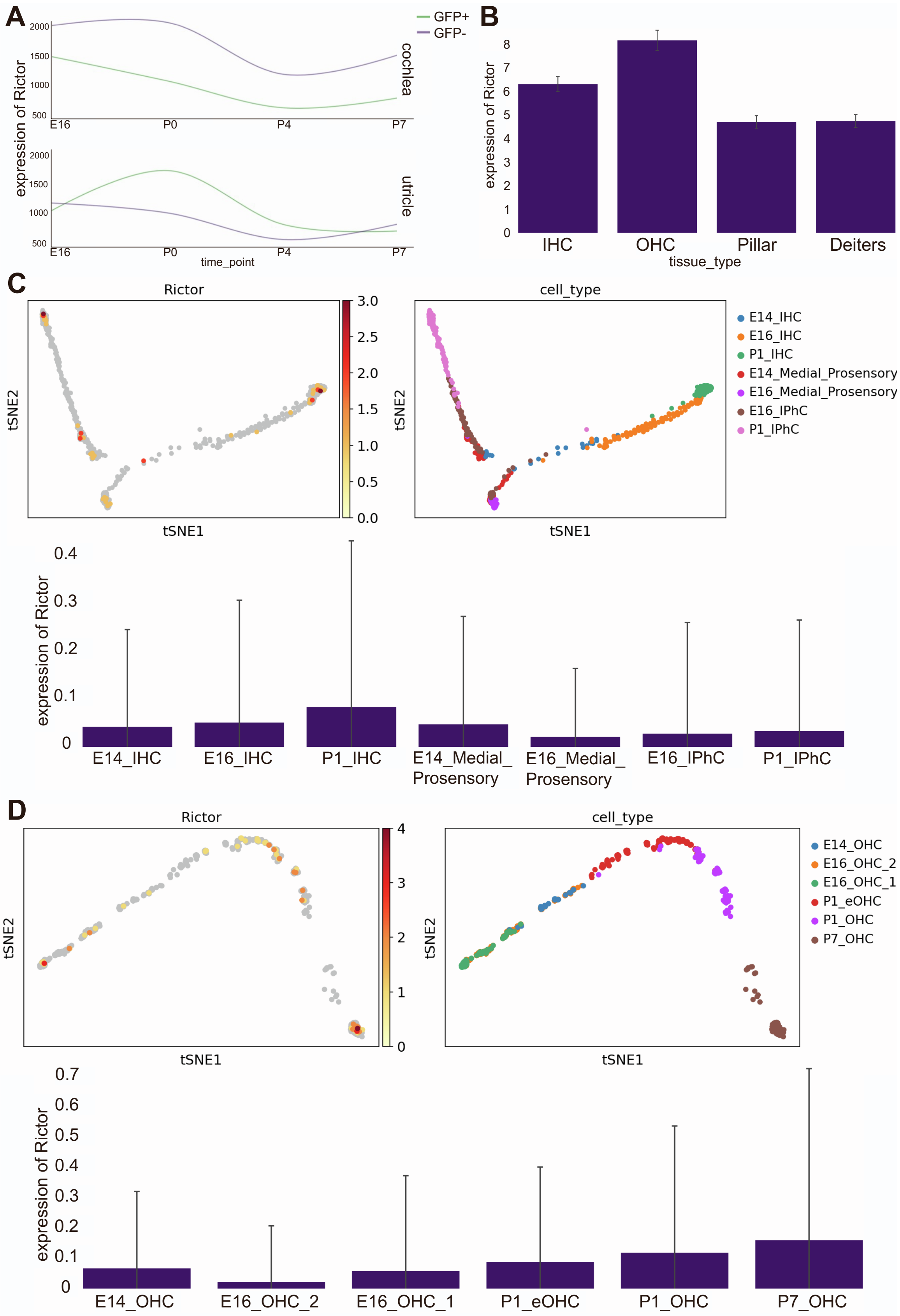
A
B
C
D
GFP+
GFP-
cochlea
utricle
expression of Rictor
time_point
E16
P0
P4
P7
tissue_type
IHC
OHC
Pillar
Deiters
Rictor
cell_type
tSNE1
tSNE2
E14_IHC
E16_IHC
P1_IHC
E14_Medial_Prosensory
E16_Medial_Prosensory
E16_IPhC
P1_IPhC
E14_Medial_ Prosensory
E16_Medial_ Prosensory
E14_OHC
E16_OHC_2
E16_OHC_1
P1_eOHC
P1_OHC
P7_OHC

Figure S1. *Rictor* expression during development, early postnatal, and adult stages in the inner ear, related to Figure 1

(A) *Rictor* gene expression in hair cells (GFP+) versus other cell types (GFP-) of the auditory and vestibular sensory epithelium. Hair cells were isolated via FACS from Pou4f3-GFP mice together with the remaining cells from dissected auditory and vestibular sensory organs at the timepoints indicated. Gene expression was measured by RNA-seq. Figure created with umgear.org [S1] with dataset from Scheffer et al. [S2].

(B) *Rictor* gene expression in cochlear hair cells (inner hair cells = IHC, outer hair cells = OHC) as well as supporting cells (pillar and Deiter's cells). 1000 cells per cell type were collected from adult (28–35 days old) CBA/J mice using the suction pipette technique. RNA was isolated from the collected cells and analyzed with RNAseq. *Rictor* expression was found in all selected cell types. Figure created with umgear.org [S1] with dataset from Liu et al. [S3].

(C) *Rictor* gene expression increases in inner hair cells (IHC) during development. Figure displays developmental expression of *Rictor* in IHCs, medial prosensory cells and inner phalangeal cells (IPhC). scRNAseq dataset from 1213 hair cells and supporting cells from the cochlear floor epithelial duct. E14 n = litter from 2 female pregnant CD1s (354 cells), E16 n = litter from 3 female pregnant CD1s (409 cells), and P1 n = 20-32 CD1 pups (450 cells). tSNE plots and bar graphs were created with umgear.org [S1] with dataset from Kolla et al. [S4].

(D) *Rictor* gene expression increases during postnatal development in outer hair cells (OHC). Figure displays developmental expression of *Rictor* in OHCs. scRNAseq dataset from 1053 OHCs from the cochlear floor epithelial duct. E14 n = litter from 2 female pregnant CD1s (34 cells), E16 n = litter from 3 female pregnant CD1s (468 cells), P1 n = 20-32 CD1 pups (371 cells), and P7 n = 15-24 CD1 pups (180 cells). OHC_1 = more mature developing OHC at E16. OHC_2 = less mature developing OHC at E16. eOHC = less mature developing OHC at P1. tSNE plots and bar graphs were created with umgear.org [S1] with dataset from Kolla et al. [S4].

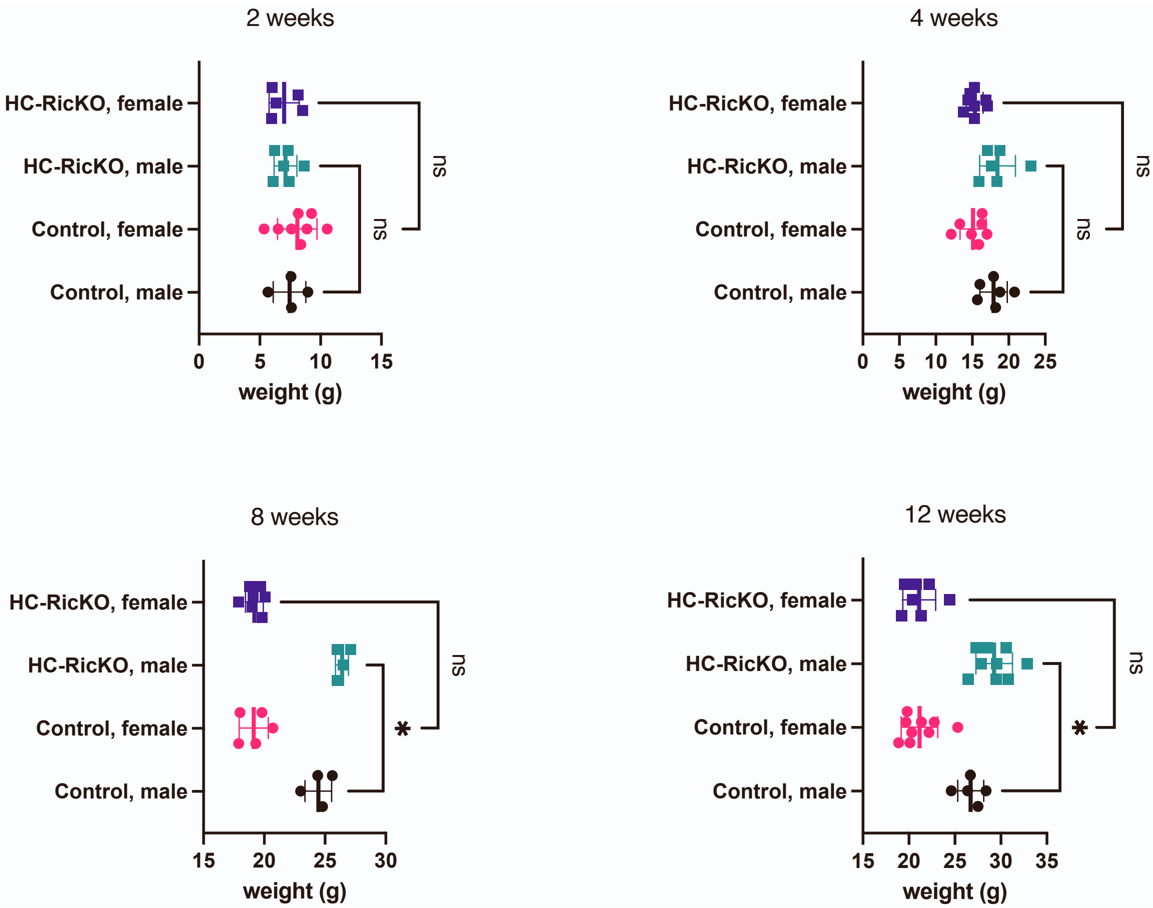

Figure S2. HC-RicKO mice have no major body weight differences when compared to littermate controls of the same sex, related to Figure 1

Weight comparisons between control and HC-RicKO mice of the same sex at timepoints indicated. There were no major weight differences between genotypes, although male HC-RicKO mice were significantly heavier than their littermate controls at 8 and 12 weeks of age. 2 weeks n = 4-6 males and 5-8 females, 4 weeks n = 6 males and 7-8 females, 8 weeks n = 4 males and 5-7 females, 12 weeks 5-9 males and 7-9 females per genotype. Results are presented as means ± SDs. Student's t-test, ns not significant, *p < 0.05.

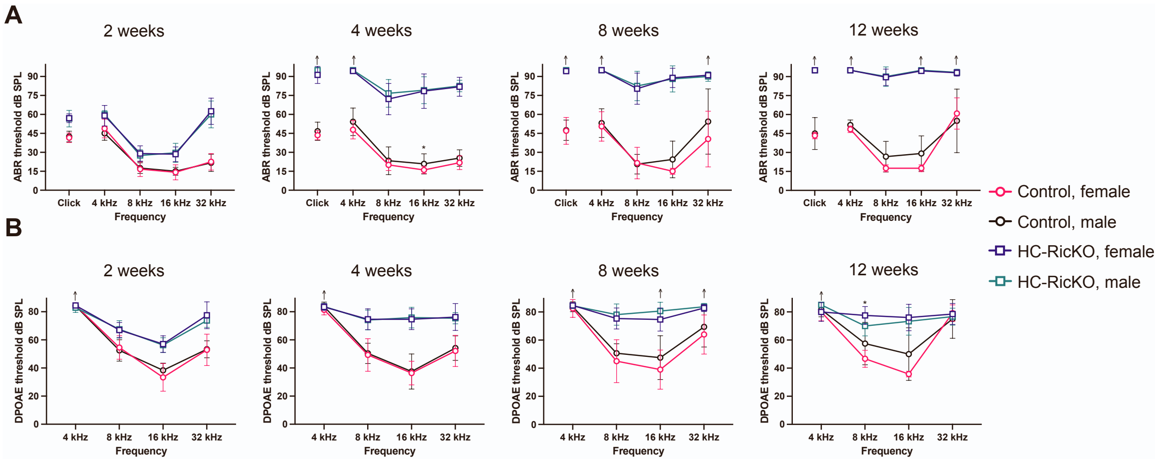

Figure S3. No differences in ABR or DPOAE hearing thresholds between male and female HC-RicKO or control mice, related to Figure 2

(A) No differences in ABR thresholds between male and female mice of the same genotype. ABR thresholds measured for a click or pure-tone stimulus at frequencies indicated at 2 weeks (control n = 6-13 ears from 3-7 animals; HC-RicKO n = 10-12 ears from 5-6 animals), 4 weeks (control n = 12-14 ears from 6-7 animals; HC-RicKO n = 12-16 ears from 6-8 animals), 8 weeks (control n = 8-10 ears from 4-5 animals; HC-RicKO n = 8-14 ears form 4-7 animals), and 12 weeks of age (control n = 6 ears from 3 animals; HC-RicKO n = 6-10 ears form 3-5 animals). The arrows indicate that even at the highest SPL level tested (90 dB SPL) there was no response. Results are presented as means ± SDs. Student's t-test, *p < 0.05, all other sex comparisons of the same genotype not significant.

(B) No differences in DPOAE thresholds between male and female mice of the same genotype. DPOAE thresholds measured for frequencies indicated at 2 weeks (control n = 6-12 ears from 3-7 animals; HC-RicKO n = 10-12 ears from 5-6 animals), 4 weeks (control n = 12-14 ears from 6-7 animals; HC-RicKO n = 12-16 ears from 6-8 animals), 8 weeks (control n = 8-10 ears from 4-5 animals; HC-RicKO n = 8-14 ears form 4-7 animals), and 12 weeks of age (control n = 6 ears from 3 animals; HC-RicKO n = 6-10 ears form 3-5 animals). The arrows indicate that even at the highest SPL level tested (80 dB SPL) there was no response. Results are presented as means ± SDs. Student's t-test, *p < 0.05, all other sex comparisons of the same genotype not significant.

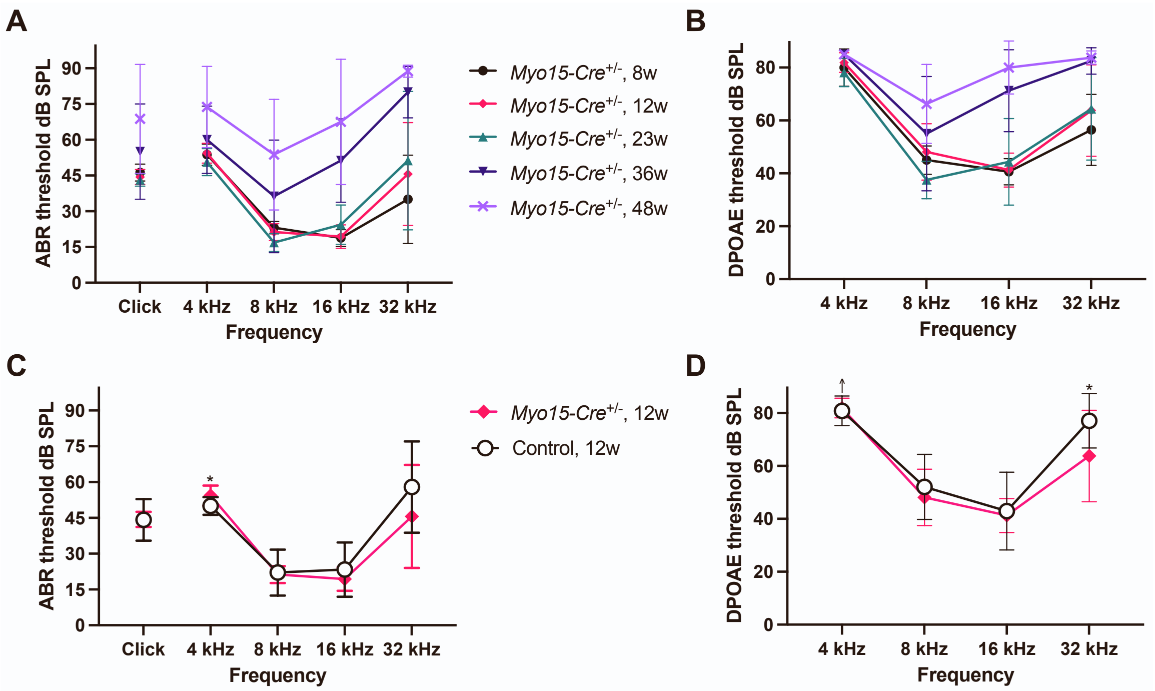

Figure S4. *Myo15-Cre*$^{+/-}$ and *Rictor*$^{fl/fl}$ control mice have similar ABR and DPOAE hearing thresholds, related to Figure 2

(A) ABR hearing thresholds in *Myo15-Cre*$^{+/-}$ mice aged 8-48 weeks measured for a click stimulus or frequencies indicated. n= 8 ears from 4 animals (8-23 weeks) and 4 ears from 2 animals (36-48 weeks). Results are presented as means ± SDs.

(B) DPOAE hearing thresholds in *Myo15-Cre*$^{+/-}$ mice aged 8-48 weeks measured for frequencies indicated and ears/animals listed in (A). Results are presented as means ± SDs.

(C) ABR hearing thresholds in *Myo15-Cre*$^{+/-}$ mice compared to *Rictor*$^{fl/fl}$ control mice at 12 weeks of age. Stimuli were either a click stimulus or pure-tones at frequencies indicated. n= 8-12 ears from 4-6 animals. Results are presented as means ± SDs. Student's t-test, *p < 0.05 at 4kHz, not significant for all other stimuli.

(D) DPOAE hearing thresholds in *Myo15-Cre*$^{+/-}$ mice compared to *Rictor*$^{fl/fl}$ control mice at 12 weeks of age measured for frequencies indicated. The arrow indicates that even at the highest SPL level tested (80 dB SPL) there was no response. n= 8-12 ears from 4-6 animals. Results are presented as means ± SDs. Student's t-test, *p < 0.05 at 32kHz, not significant for all other stimuli.

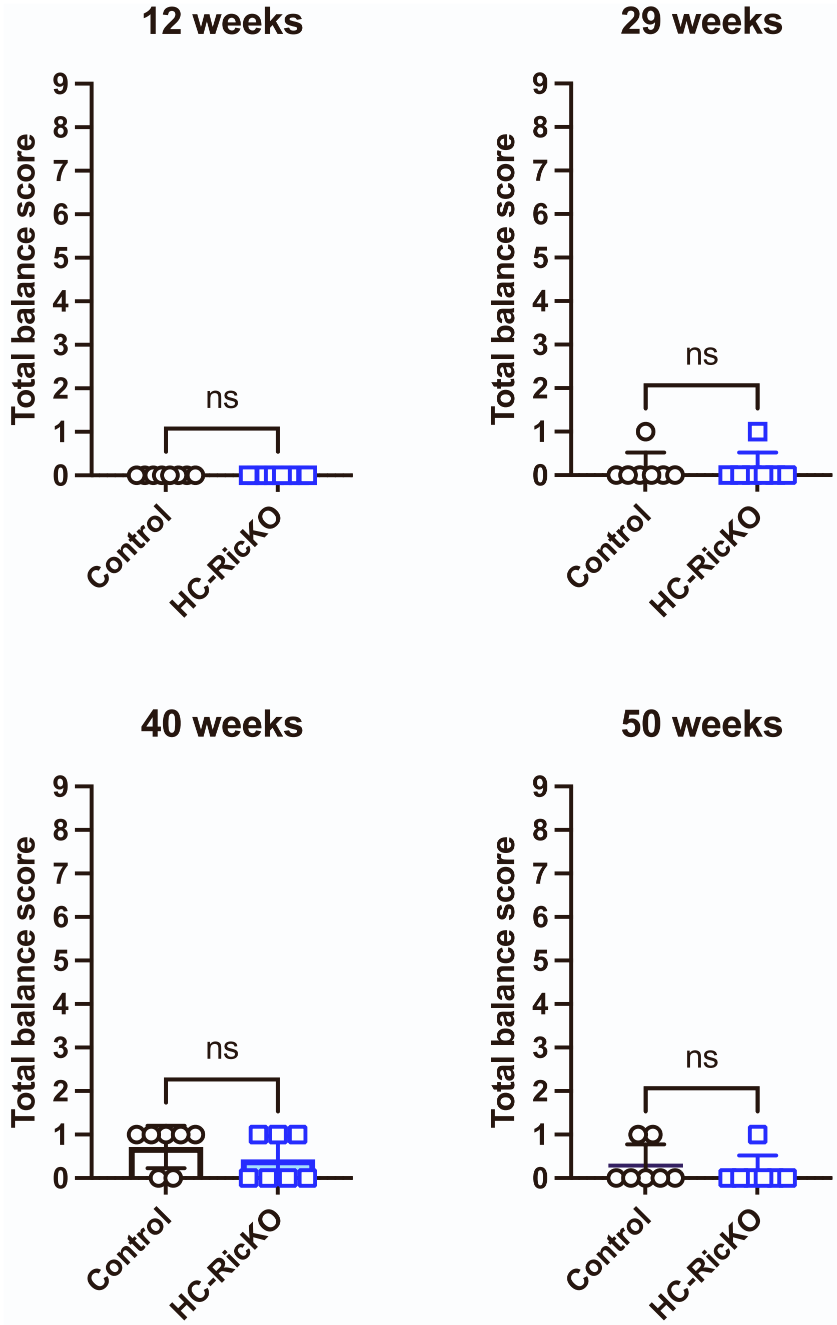

Figure S5. HC-RicKO mice have normal vestibular function until advanced age when assessed with a behavioral vestibular phenotyping pipeline, related to Figure 2

HC-RicKO mice have normal vestibular function similar to control mice until advanced age. A phenotyping pipeline was used to analyze vestibular function. A score (0-9) was calculated by monitoring head tossing or circling behavior and performing a trunk curl test, a contact righting test, and a swim test. A score of 0 represents a normal vestibular function, whereas a score of 9 represents severe vestibular dysfunction. n = 7-8 mice per genotype. Results are presented as means ± SDs. Mann-Whitney test, ns not significant.

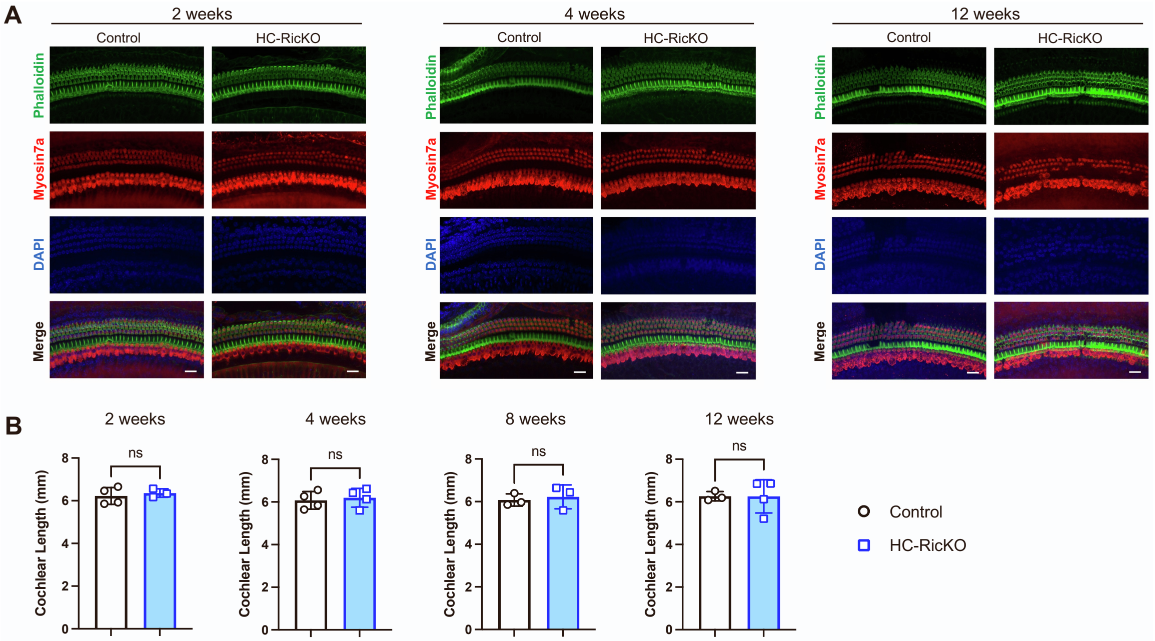

Figure S6. Hearing loss precedes hair cell loss in HC-RicKO mice, related to Figure 3

(A) Representative images (maximum intensity projections) of the medial cochlear turn from mice at indicated ages. Hair cells are visualized with phalloidin, a Myosin7a antibody and nuclear DAPI staining. Scale bar for all figures = 20μm.

(B) Cochlear length measured along the inner hair cell lateral margin from mice at indicated ages. n = 3-4 mice (2 weeks), 4 mice (4 weeks), 3 mice (8 weeks), 3-4 mice (12 weeks). Results are presented as means ± SDs. Student's t-test, ns not significant.

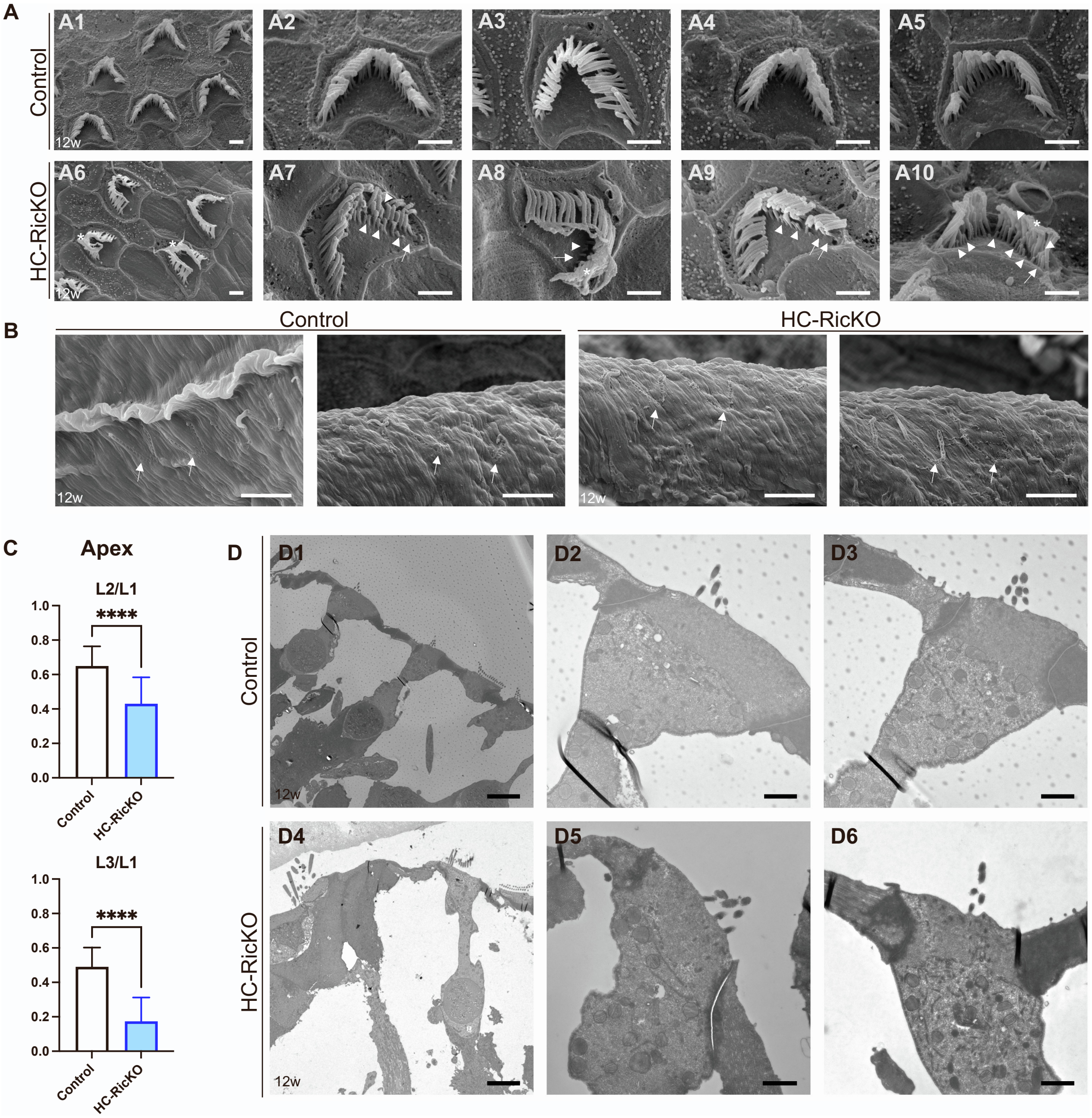

Figure S7. HC-RicKO mice show abnormal OHC stereocilia but intact tectorial membrane imprints and normal cell morphology, related to Figure 4

(A) SEM images of control (A1-A5) and HC-RicKO (A6-A10) outer hair cell (OHC) stereocilia. Stereocilia from OHCs of HC-RicKO mice show an amorphous structure on the outermost row (asterisk), shortened stereocilia (arrowheads), and missing stereocilia (arrows). All images are representative images from the medial cochlear turn of 12 week old mice. n = 3 mice per genotype. Scale bar for all figures = 1μm.

(B) The outermost stereocilia row of OHCs directly inserts in the tectorial membrane. The tectorial membrane of both control and HC-RicKO mice shows imprints of stereocilia (arrows) on the lower surface. Images from 12 week old mice. n = 3 mice per genotype. Scale bar for all figures = 3μm.

(C) Analysis of OHC stereocilia length in the apical cochlear turn, where the length from the middle (L2) and small (L3) stereocilia rows was normalized to the length of stereocilia in the tall row (L1). There was no significant difference in the length of stereocilia from the tallest row (L1) between

genotypes. 6-8 cells from 3 mice were analyzed per genotype. Results are presented as means ± SDs. Student's t-test, ****p < 0.0001.

(D) TEM images of control (D1-D3) and HC-RicKO (D4-D6) outer hair cells. HC-RicKO hair cells show normal cellular morphology comparable to control mice. All images are from 12 week old mice. n = 3 mice per genotype. Scale bar D1 and D4 = 5μm; D2, D3, D5, and D6 = 1μm.

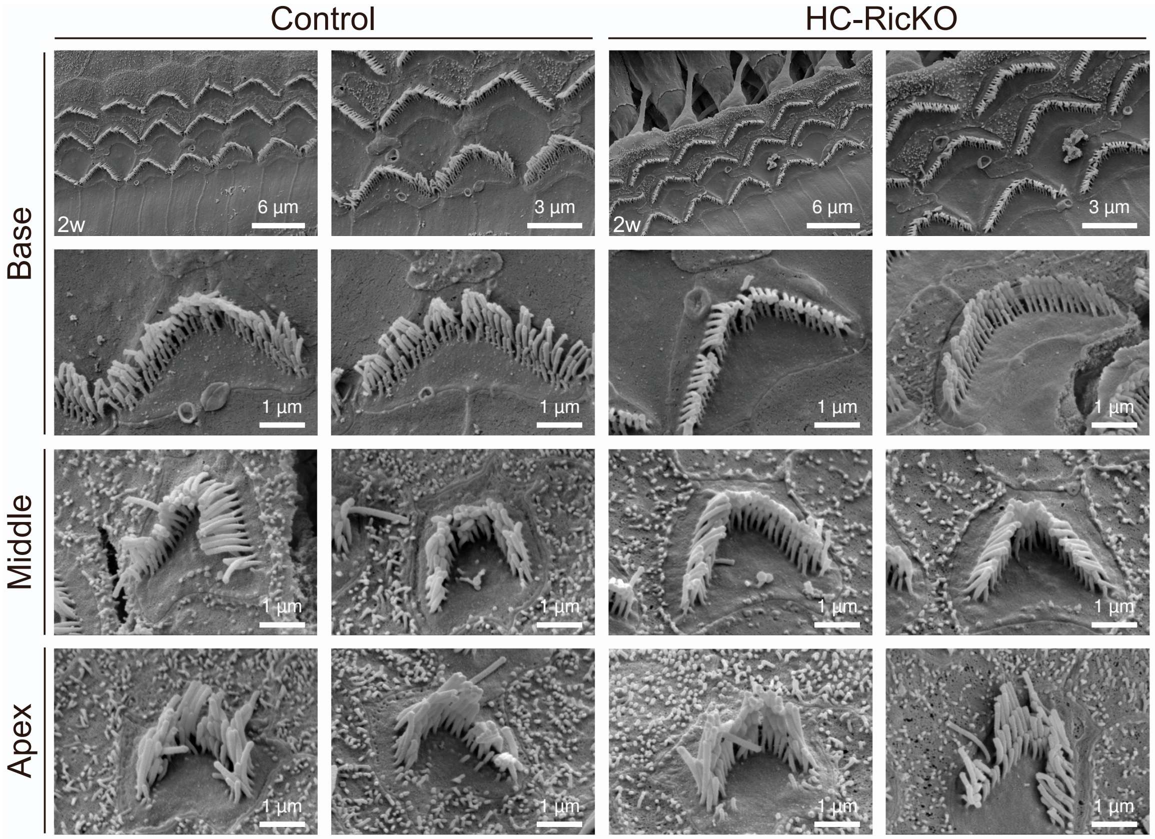

Figure S8. HC-RicKO mice have normal stereocilia development, related to Figure 4

SEM images of control and HC-RicKO outer hair cell (OHC) stereocilia at 2 weeks of age (P14) from different cochlear regions. Stereocilia from HC-RicKO mice develop normally and are similar to those in control mice. n = 3 mice per genotype. Scale bar sizes are indicated in the corresponding figure panels.

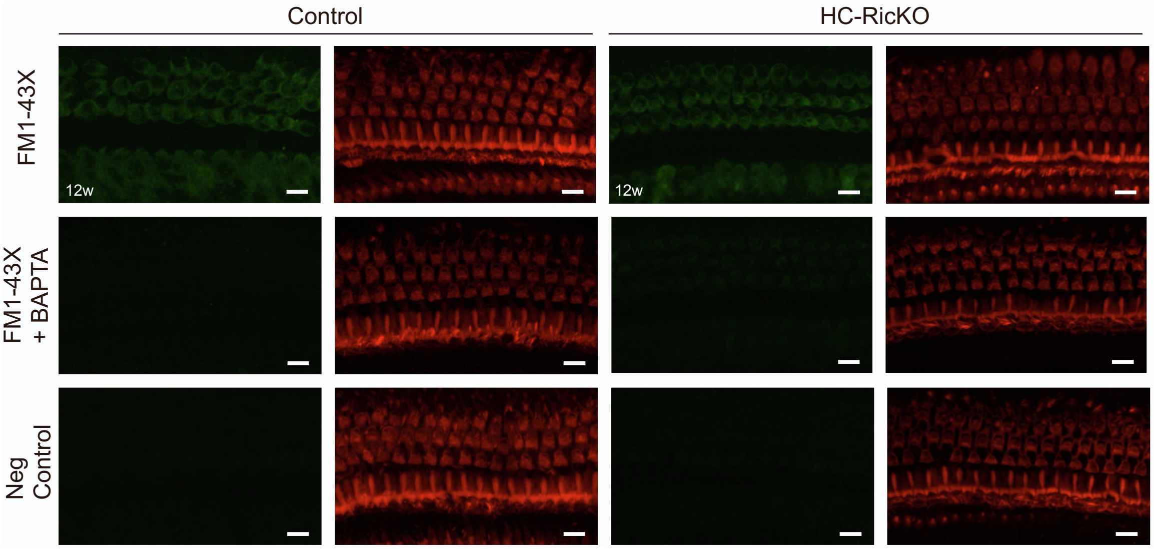

Figure S9. No difference in FM1-43X uptake between control and HC-RicKO mice in the Apex, related to Figure 4

FM1-43X *in situ* staining of adult cochlear hair cells. Images are maximum intensity projections from the apical cochlear turn. Cochleae of 12 week old mice were either perfused with FM1-43X (green), FM1-43X + BAPTA or HBSS only for the negative control (Neg Control). To identify the hair cells, actin filled stereocilia and cuticular plates were stained with phalloidin (red). Data from n = 3 mice for FM1-43X and 2 mice for FM1-43X + BAPTA staining. Scale bar for all figures = 10μm.

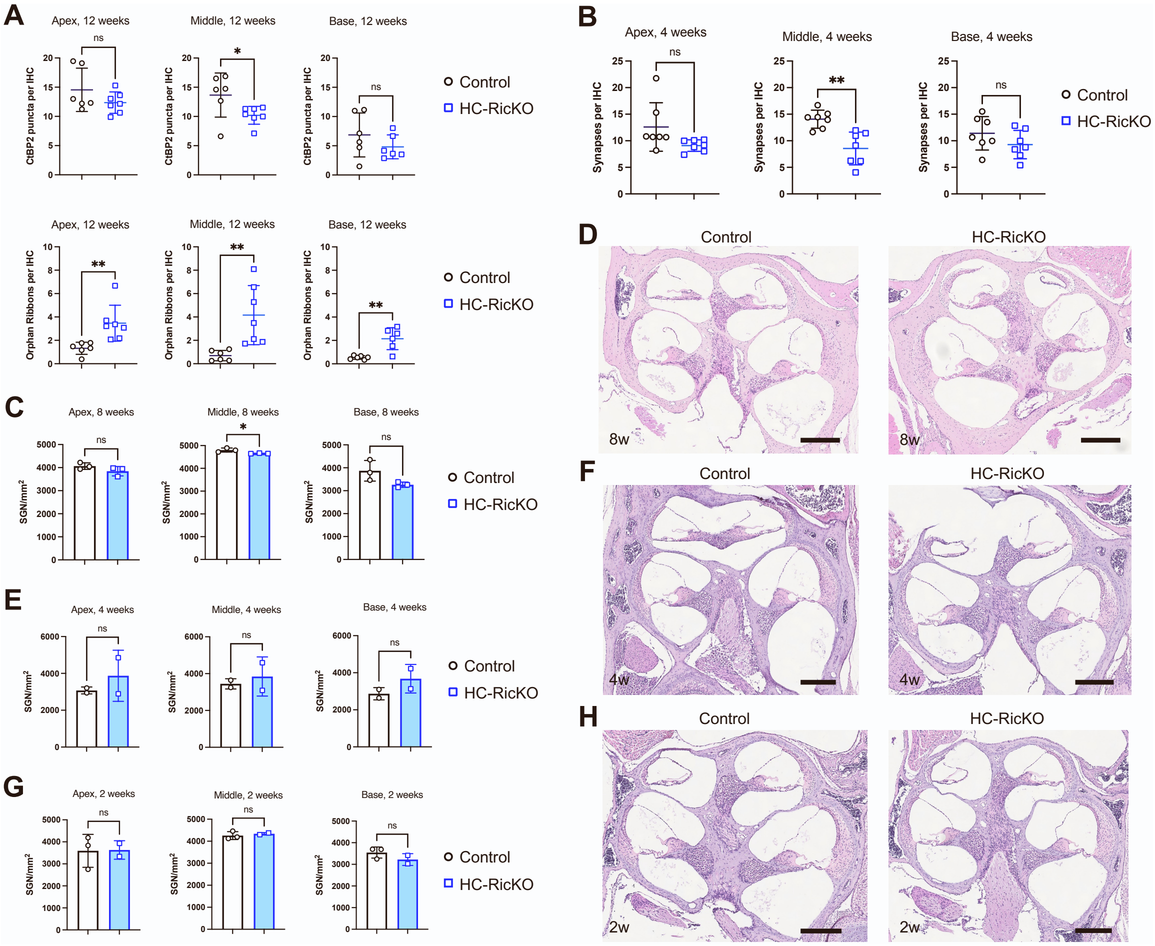

Figure S10. HC-RicKO mice show increased numbers of orphan ribbons, reduced synapse numbers but normal spiral ganglion neuron counts, related to Figure 5

(A) Quantification of the total number of CtBP2 puncta as well as orphan ribbons (CtBP2 puncta not juxtaposed to GluR2) per inner hair cell (IHC) in 12 week old mice. Data from n = 6-7 ears from 4-5 animals. Results are presented as means ± SDs. Student's t-test, *p < 0.05, **p < 0.01, ns not significant.

(B) Quantification of synapse counts (juxtaposed CtBP2-GluR2) per inner hair cell (IHC) in 4 week old mice. Data from n = 7 ears from 5 animals. Results are presented as means ± SDs. Student's t-test, **p < 0.01, ns not significant.

(C) Quantification of SGN counts in H&E stained histological sections of 8 week old mice. Data from n = 3 mice. Counts were normalized to area (SGN/mm^2) and averaged for each cochlear turn of the same ear before analysis. Results are presented as means ± SDs. Student's t-test, *p < 0.05, ns not significant.

(D) Midmodiolar cochlear sections stained with H&E from 8 week old mice. Scale bar for all figures = 250µm.

(E) Quantification of SGN counts in H&E stained histological sections of 4 week old mice. Data from n = 2 mice. Counts were normalized to area (SGN/mm^2) and averaged for each cochlear turn

of the same ear before analysis. Results are presented as means ± SDs. Student's t-test, ns not significant.

(F) Midmodiolar cochlear sections stained with H&E from 4 week old mice. Scale bar for all figures = 250μm.

(G) Quantification of SGN counts in H&E stained histological sections of 2 week old mice. Data from n = 2-3 mice. Counts were normalized to area (SGN/mm^2) and averaged for each cochlear turn of the same ear before analysis. Results are presented as means ± SDs. Student's t-test, ns not significant.

(H) Midmodiolar cochlear sections stained with H&E from 2 week old mice. Scale bar for all figures = 250μm.

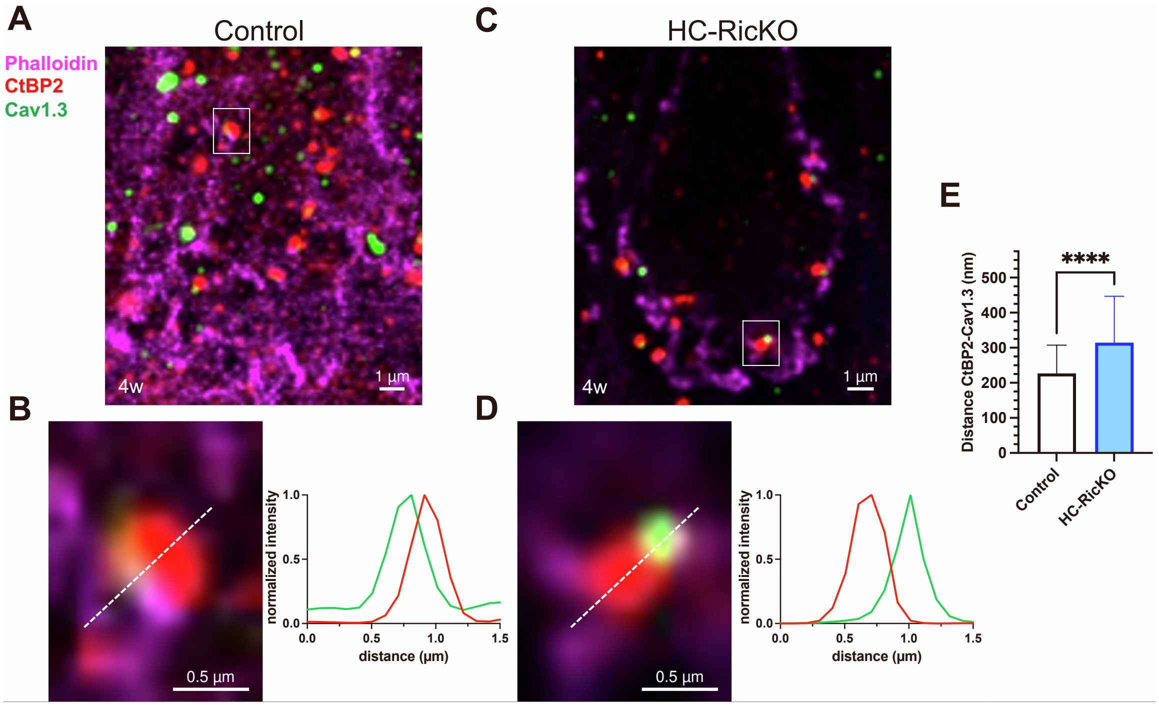

Figure S11. HC-RicKO mice show a reduced synaptic F-actin network in inner hair cells at 4 weeks of age, related to Figure 6

(A) Representative image (maximum intensity projection, 7 z-layers of 0.125μm each) of an inner hair cell from the medial cochlear turn of a 4 week old control mouse. Phalloidin labelled F-actin (magenta) forms a network surrounding ribbon synapses (CtBP2) and calcium channels (Cav1.3). Magnification of the inset is displayed in panel (B). Scale bar size = 1μm.

(B) Magnification from the square shown in panel (A). The ribbon synapse (CtBP2, red) lies in proximity of the calcium channel (Cav1.3, green) in control mice. The graph represents the fluorescent intensity profile of the synapse measured along the white dashed line. Scale bar size = 0.5μm.

(C) Representative image (maximum intensity projection, 7 z-layers of 0.125μm each) of an inner hair cell from the medial cochlear turn of a 4 week old HC-RicKO mouse. Phalloidin labelled F-actin (magenta) shows a cortical, submembranous localization. Magnification of the inset is displayed in panel (D). Scale bar size = 1μm.

(D) Magnification from the square shown in panel (C). There is a larger distance between the ribbon synapse (CtBP2, red) and calcium channel (Cav1.3, green) in HC-RicKO mice. The graph represents the fluorescent intensity profile of the synapse measured along the white dashed line. Scale bar size = 0.5μm.

(E) Quantification of the center mass distance between ribbon (CtBP2) and Cav1.3. Data from n = 5 ears from 3 animals aged 4 weeks, where 275 active zones per genotype were analyzed. Results are presented as means + SDs. Student's t-test, ****$p < 0.0001$.

Table S1. HC-RicKO mice have normal vestibular function at 12 weeks of age when assessed with a behavioral vestibular phenotyping pipeline, related to Figure 2

**Mouse n°**	**Age**	**Sex**	**Genotype**	**Total balance scoring**	**Head tossing**	**Circling**	**Trunk curl test**	**Contact righting test**	**Swimming test**
	weeks		*Myo15-Cre*<br>- Cre/wt or<br>- wt/wt<br><br>all are *Rictor*$^{fl/fl}$		**0 = no<br>1 = yes**	**0 = no<br>1 = yes**	**0 = normal<br>1 = deficient**	**0 = immediately rotate back to their upright position<br>1 = no rotation, slowly moves<br>2 = inverted for at least 30s<br>3 = remain inverted**	**0 = swims<br>1 = irregular swimming<br>2 = immobile floating<br>3 = underwater tumbling**
1	12	f	wt/wt	**0**	0	0	0	0	0
2	12	f	wt/wt	**0**	0	0	0	0	0
3	12	f	wt/wt	**0**	0	0	0	0	0
4	11	f	wt/wt	**0**	0	0	0	0	0
5	11	f	wt/wt	**0**	0	0	0	0	0
6	11	f	wt/wt	**0**	0	0	0	0	0
7	11	m	wt/wt	**0**	0	0	0	0	0
8	11	m	wt/wt	**0**	0	0	0	0	0
9	12	f	Cre/wt	**0**	0	0	0	0	0
10	11	f	Cre/wt	**0**	0	0	0	0	0
11	12	m	Cre/wt	**0**	0	0	0	0	0
12	12	m	Cre/wt	**0**	0	0	0	0	0
13	12	m	Cre/wt	**0**	0	0	0	0	0
14	12	m	Cre/wt	**0**	0	0	0	0	0
15	11	m	Cre/wt	**0**	0	0	0	0	0
16	11	m	Cre/wt	**0**	0	0	0	0	0

SI References

S1. Orvis, J., Gottfried, B., Kancherla, J., Adkins, R.S., Song, Y., Dror, A.A., Olley, D., Rose, K., Chrysostomou, E., Kelly, M.C., et al. (2021). gEAR: Gene Expression Analysis Resource portal for community-driven, multi-omic data exploration. Nat Methods *18*, 843-844. 10.1038/s41592-021-01200-9.

S2. Scheffer, D.I., Shen, J., Corey, D.P., and Chen, Z.Y. (2015). Gene Expression by Mouse Inner Ear Hair Cells during Development. J. Neurosci. *35*, 6366-6380. 10.1523/JNEUROSCI.5126-14.2015.

S3. Liu, H., Chen, L., Giffen, K.P., Stringham, S.T., Li, Y., Judge, P.D., Beisel, K.W., and He, D.Z.Z. (2018). Cell-Specific Transcriptome Analysis Shows That Adult Pillar and Deiters' Cells Express Genes Encoding Machinery for Specializations of Cochlear Hair Cells. Front. Mol. Neurosci. *11*, 356. 10.3389/fnmol.2018.00356.

S4. Kolla, L., Kelly, M.C., Mann, Z.F., Anaya-Rocha, A., Ellis, K., Lemons, A., Palermo, A.T., So, K.S., Mays, J.C., Orvis, J., et al. (2020). Characterization of the development of the mouse cochlear epithelium at the single cell level. Nat Commun *11*, 2389. 10.1038/s41467-020-16113-y.
